# Emerging Computational Advances in Simulating PCSK9 Variant Dynamics for Therapeutic Design Against Hypercholesterolemia

**DOI:** 10.3390/ijms27219676

**Published:** 2026-10-29

**Authors:** Ma. Cecilia Opazo, Andrés Gallegos, Antar Muñoz, Roger Zapata, Juan Carlos Rivera, Romina V. Sepúlveda, Daniel Aguayo

**Affiliations:** 1 Facultad de Medicina Veterinaria y Agronomía, Instituto de Ciencias Naturales, Universidad de Las Américas, Santiago 7500975, Chile; mopazod@udla.cl; 2 Centro de Investigación en Ciencias Biológicas y Químicas, Universidad de Las Américas, Santiago 7500975, Chile; andres.gallegospinto@gmail.com (A.G.); antar.munoz@gmail.com (A.M.); 3 Programa de Magister en Ciencias Biológicas y Químicas, Facultad de Medicina Veterinaria y Agronomía, Instituto de Ciencias Naturales, Universidad de las Américas, Santiago 7500000, Chile; 4 Departamento de Ciencias de la Ingeniería, Facultad de Ingeniería, Universidad Andrés Bello, Santiago 8370146, Chile; r.zapatatoledo@uandresbello.edu; 5 Institute for Advanced Biomedical Research, Charles R. Drew University of Medicine and Science, Los Angeles, CA 90059, USA; juancarlosriveracam@cdrewu.edu; 6 Center for Bioinformatics and Integrative Biology (CBIB), Facultad de Ciencias de La Vida, Universidad Andres Bello, Santiago 8370146, Chile; romina.sepulveda@unab.cl; 7 ANID–Millenium Science Initiative Program–Millenium Nucleus in Data Science for Plant Resilience (PhytoLearning), Santiago 8370186, Chile

**Keywords:** PCSK9, molecular dynamics simulation, gain-of-function and loss-of-function variants, cysteine-histidine-rich domain (CHRD), generative protein design

## Abstract

Proprotein convertase subtilisin/kexin type 9 (PCSK9) is a premier model for precision medicine, having moved from genetic discovery to approved therapeutics within two decades. Yet despite the clinical success of monoclonal antibodies, small interfering RNA and the newly approved oral macrocyclic-peptide inhibitor class, targeting PCSK9 remains a demanding biophysical challenge. Clinical translation has concentrated almost entirely on the rigid, well-resolved subtilisin-like catalytic domain that interfaces with the low-density lipoprotein receptor (LDLR). By contrast, the C-terminal cysteine- and histidine-rich domain (CHRD), which governs allosteric regulation, protein trafficking and post-translational modification, is highly disordered and largely elusive in experimental structures, leaving a critical regulatory network therapeutically unexploited. This review examines how advanced computational biology is bridging that structural gap, organizing the computational landscape of PCSK9 around the biophysical mechanisms of gain- and loss-of-function variants. We detail how explicit-solvent molecular dynamics, free-energy calculations and cryptic-pocket detection expose the functional plasticity and allosteric networks that static crystallography cannot capture, and we evaluate the convergence of these physics-based approaches with artificial intelligence, including deep-learning structure prediction and generative design of de novo nanobody and peptide binders. Therapeutic development and the structural literature currently point in diverging directions: drug discovery remains focused on the crowded LDLR-binding interface, whereas computational methods are beginning to identify candidate sites beyond it. We argue that the next advance in PCSK9 therapeutics will come from integrating generative artificial intelligence with rigorous enhanced sampling in a unified pipeline, deliberately shifting focus toward the disordered regulatory domains that have evaded classical screening.

## 1. Introduction

**Figure 1:**
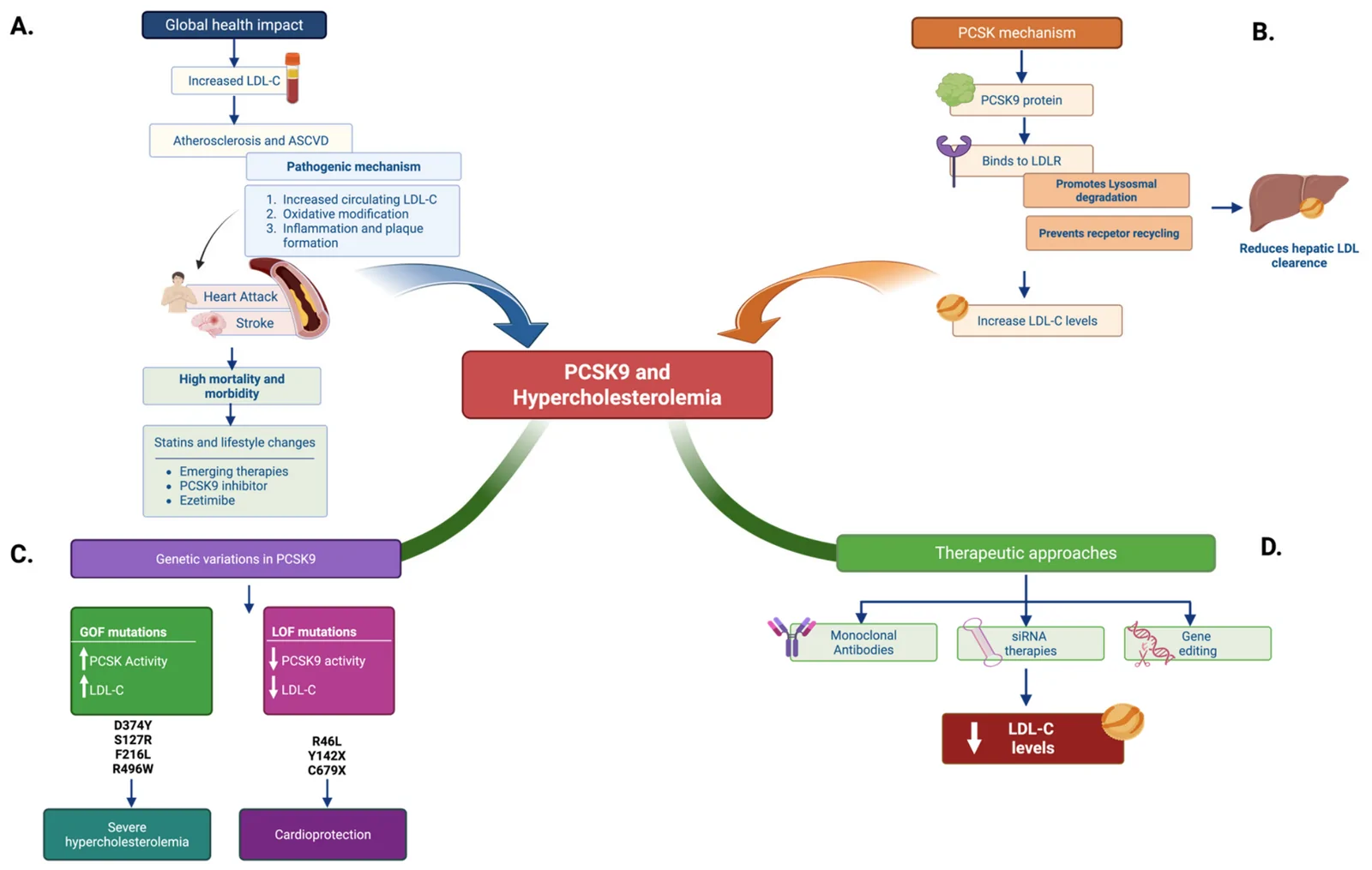
The role of PCSK9 in LDL-C regulation, genetic disease, and targeted therapies. (**A**) Schematic overview linking elevated low-density lipoprotein cholesterol (LDL-C) to atherosclerosis and atherosclerotic cardiovascular disease (ASCVD), culminating in major clinical events (myocardial infarction and stroke) and increased morbidity and mortality. The central panel highlights PCSK9 and hypercholesterolemia as an integrating concept. (**B**) At the mechanistic level, secreted PCSK9 binds the LDL receptor (LDLR), promoting increased circulating LDL-C. (**C**) Genetic effects of PCSK9 variation are summarized: gain-of-function (GOF) variants increase PCSK9 activity and raise LDL-C, whereas loss-of-function (LOF) variants reduce PCSK9 activity and lower LDL-C. (**D**) Therapeutic strategies targeting this axis are shown, including monoclonal antibodies, siRNA-based therapies, and gene editing, all converging on reduced LDL-C levels. The left pathway also contextualizes standard-of-care and adjunct lipid-lowering interventions (statins/lifestyle; emerging therapies such as ezetimibe and PCSK9 inhibitors). Created in https://BioRender.com.

Hypercholesterolemia, characterized by elevated low-density lipoprotein cholesterol (LDL-C), continues to represent a significant global health challenge due to its pivotal role in the development of atherosclerotic cardiovascular disease (ASCVD), the foremost cause of mortality worldwide [1] (Figure 1A). The pathogenic mechanisms include increased levels of circulating LDL-C infiltrating the arterial intima, undergoing oxidative modification and leading to endothelial dysfunction, persistent inflammation, and plaque formation; these processes accelerate atherosclerosis and heighten the risk of myocardial infarction and stroke [2] (Figure 1A). While lifestyle interventions and high-intensity statins remain standard first-line treatments, a considerable subset of patients does not achieve recommended LDL-C targets, revealing ongoing challenges in risk reduction [3].

Identified in 2003 as the third major gene associated with autosomal dominant familial hypercholesterolemia [4], proprotein convertase subtilisin/kexin type 9 (PCSK9) is now acknowledged as a key extracellular regulator of cholesterol homeostasis due to its influence on hepatic LDL receptor (LDLR) abundance, the principal pathway for removing circulating low-density lipoprotein cholesterol (LDL-C) [5] (Figure 1B). Secreted PCSK9 binds the LDL receptor and directs it toward lysosomal degradation, thereby raising circulating LDL-C [6]; the molecular basis of this interaction and its transcriptional regulation by the SREBP-2 sterol-sensing network are detailed in Section 2.1.

Consistent findings from both human studies and animal models demonstrate that loss-of-function (LOF) variants in PCSK9 result in increased hepatic LDLR and significantly reduced LDL-C, whereas gain-of-function (GOF) variants enhance LDLR degradation and raise LDL-C. These observations confirm that PCSK9 exerts its primary physiological effects on cholesterol balance via regulation of hepatic LDLR [5,7]. Recent analyses [8,9] indicate that cardiovascular diseases associated with high LDL-C account for millions of deaths and disability-adjusted life years, underscoring dyslipidemia as a widespread risk factor across varied populations.

The present review is deliberately narrow in focus: we examine how simulation and artificial intelligence are being used to interpret disease-associated PCSK9 variants and to identify therapeutically tractable surfaces beyond the canonical LDLR-binding interface. Section 2 first consolidates the domain architecture, LDLR-engagement mechanism, transcriptional and post-translational regulation, and structural genetics of PCSK9, and then surveys the bioinformatic, molecular-dynamics and machine-learning methods that have been applied to this target. Section 3 examines gain- and loss-of-function variants in monogenic disease, Section 4 considers the clinical and therapeutic implications, and Section 5 sets out the open questions and the research priorities we regard as most tractable.

## 2. Computational and Bioinformatic Characterization of Pcsk9 Variants

### 2.1. Pcsk9 Domain Architecture, LDLR Engagement and Regulation

The PCSK9 gene, composed of 12 exons over approximately 3.7 kb of mRNA, is located on chromosome 1p32 and encodes a secreted protein consisting of 692 amino acids [10,11]. PCSK9 is initially synthesized as a zymogen and subsequently undergoes autocatalytic cleavage in the early endoplasmic reticulum at the VFAQ↓152 site. After this processing event, the excised pro domain remains closely associated with the catalytic domain, effectively blocking “protease-in-trans” activity and accounting for PCSK9’s predominantly non-enzymatic, stoichiometric effect on LDLR, rather than direct substrate proteolysis [5]. This distinction has direct therapeutic implications. Because PCSK9 acts as a chaperone rather than a protease toward LDLR, effective inhibition does not require blocking a catalytic site, only preventing the PCSK9–LDLR interaction itself, which is precisely the mechanism exploited by both antibody-based neutralization and hepatic-silencing therapies rather than classical enzyme-inhibitor design.

Structural characterization of PCSK9 has relied predominantly on X-ray crystallographic studies, conducted under neutral and acidic conditions, which have revealed a multi-domain architecture. On the other hand, cryo-electron microscopy has not yet yielded a deposited PCSK9 structure, though it is an increasingly relevant complementary approach as deposited structures of PCSK9-partner assemblies with LDLR or antibody Fabs grow larger and more conformationally heterogeneous. Structurally, PCSK9 features several distinct domains: a signal peptide (amino acids 1–30), a pro domain (amino acids ~31–152) functioning as both intramolecular chaperone and inhibitor essential for ER export, a catalytic domain (amino acids ~153–421) that serves as the primary interface with the LDL receptor, particularly its EGF-A domain. The catalytic domain itself harbors the subtilisin-like catalytic triad (Asp186, His226, Ser386) [12]. PCSK9 additionally comprises a hinge region (amino acids ~422–452) and a C-terminal cysteine/histidine-rich domain (CHRD; amino acids ~453–692), which contains three modules (M1, M2, M3) important for LDLR degradation and trafficking but not necessary for receptor binding [5,13]. The pro domain partially covers the catalytic fold, the catalytic domain constitutes the main LDLR-binding surface, and the CHRD contributes additional interaction sites involved in endosomal sorting and partner recognition. This structural framework aids in understanding how variants across different domains impact secretion, LDLR affinity, and receptor-degradation functions [13,14,15].

Building on this modular architecture, PCSK9 regulates hepatic LDL receptor (LDLR) levels through a non-proteolytic trafficking mechanism that directly influences circulating LDL-C. PCSK9 also engages nascent LDLR intracellularly during secretory trafficking, though this is a minor route; in the predominant extracellular pathway, secreted PCSK9 interacts with the EGF-A domain of LDLR at the hepatocyte surface via its catalytic domain interface. This interaction occurs at the cell surface under physiological pH conditions through an antiparallel structural arrangement stabilized by calcium-dependent salt bridges. In addition, the L108 residue located in the PCSK9 pro-domain directly contacts the L626 residue of LDLR, strengthening the initial association [12,16]. The resulting PCSK9–LDLR complex is subsequently internalized, typically through clathrin-mediated endocytosis, into endosomes. Within these compartments, the increasingly acidic environment promotes sustained association between PCSK9 and LDLR, redirecting LDLR from recycling pathways toward late endosomal/lysosomal degradation [5,17]. Effective lysosomal targeting relies not only on this binding but also on the involvement of PCSK9’s CHRD and associated partner-dependent trafficking events, which facilitate LDLR entry into degradative processes, thereby decreasing LDLR surface density and reducing hepatic clearance of LDL particles [5,13]. This process prevents LDLR from recycling back to the plasma membrane and from participating again in LDL cholesterol uptake [15]. Consequently, the levels of LDL-R on the surface are significantly reduced, maintaining high levels of LDL-C in the plasma [18], promoting the accumulation of cholesterol in the arterial wall and contributing to the development and progression of atherosclerotic cardiovascular disease [19]. Consequently, elevated PCSK9 activity, or GOF variants that increase complex stability or trafficking, results in higher plasma LDL-C concentrations. By contrast, inhibition of PCSK9 maintains LDLR recycling, enhances receptor availability for LDL uptake, and leads to significant reductions in plasma LDL-C [13]. Because LDLR availability is highly sensitive to PCSK9 levels, the latter has emerged as a critical therapeutic target. By modulating this single protein–protein interaction rather than a traditional enzymatic active site, it is possible to effectively control LDL-C across a wide physiological range.

PCSK9 expression is rigorously regulated at the transcriptional level according to cellular cholesterol conditions, with sterol regulatory element–binding protein 2 (SREBP-2) serving as the principal regulator [20]. When intracellular cholesterol levels are low, SREBP-2 becomes activated and relocates to the nucleus, where it attaches to sterol regulatory elements within the PCSK9 promoter, simultaneously inducing the transcription of both PCSK9 and LDLR [13,21]. This mechanism establishes a feedback loop that prevents excessive LDLR-mediated LDL uptake and maintains plasma LDL-C equilibrium [13,14]. In addition to SREBP-2, emerging research has identified further regulatory mechanisms, such as insulin and glucose signaling, which upregulate PCSK9 transcription via hepatocyte nuclear factor-1α (HNF-1α), thereby linking PCSK9 levels to metabolic state and insulin sensitivity [13]. Inflammatory and hormonal factors have also been recognized as significant modulators: cytokine-induced pathways and nuclear receptors like FXR and PPARs impact PCSK9 expression either directly at the promoter or indirectly through interactions with SREBP-2 signaling, thus coordinating lipid metabolism with immune and endocrine functions [14,22]. Overall, data from 2024 to 2026 confirm that PCSK9 expression is controlled by a complex regulatory framework, with SREBP-2 at its core, and with metabolic, inflammatory, and nuclear-receptor influences modulating PCSK9 output to meet systemic physiological needs, ultimately determining LDLR levels and plasma LDL-C concentrations.

Although significant advances have been made in characterizing PCSK9 transcriptional regulation and LDLR binding, a comprehensive integration of the post-translational regulatory pathways controlling its activity remains incomplete. This is particularly true regarding signaling axes that involve FAM20 family kinases, cyclase-associated protein 1 (CAP1)-mediated trafficking, and mechanisms dependent on serine phosphorylation. Regarding post-translational modifications, recent structure–function analyses highlight regulatory events such as furin cleavage within an exposed loop of the catalytic domain that directly modulate PCSK9 activity. Furthermore, modifications within the flexible N-terminal prodomain, namely tyrosine sulfation (e.g., Tyr38) and serine phosphorylation (e.g., Ser47), are now known to influence cellular trafficking, ligand interactions, and variant phenotypes [5,13,23,24]. Beyond the canonical LDLR-binding interface within the catalytic domain, recent reviews focus attention on the CHRD, notably module 3, which has emerged as a critical hub for non-canonical interactions, intracellular trafficking, and functional modulation [13,17]. The C-terminal region contains functionally significant variants and is increasingly recognized as a platform for protein–protein interactions that influence endosomal sorting and receptor fate independently of LDLR affinity, most notably through phosphorylation-dependent engagement with regulatory partners such as FAM20C and CAP1, discussed below.

Recent evidence shows the involvement of secretory-pathway kinases such as FAM20C, which preferentially phosphorylate luminal or secreted protein serine residues, as key upstream regulators capable of modulating PCSK9 conformation, stability, or interaction with adaptor proteins such as CAP1, a scaffold implicated in lipid signaling and vesicular transport [14,22]. Within this context, serine phosphorylation in flexible or C-terminal regions of PCSK9 is proposed as an essential regulatory mechanism. This phosphorylation impacts secretion efficiency, persistence of the PCSK9–LDLR complex within acidic compartments, and phenotypic outcomes of certain variants, especially those located in CHRD module 3 [13]. Overall, PCSK9 regulation is driven by a multilayered post-translational network that is not yet fully elucidated, with C-terminal serine phosphorylation and related trafficking pathways representing leading areas of research for understanding disease-associated variants and discovering novel regulatory or therapeutic targets.

This regulatory complexity is matched by commercial momentum. Although commercial market-research projections vary across vendors and lack peer review, current estimates place the global PCSK9-inhibitor market at roughly US $3–4 billion in 2025–2026, with a projected double-digit compound annual growth rate over the following decade [25]. Furthermore, the clinical pipeline extends well beyond the approved antibodies and siRNA discussed above, now including investigational oral small-molecule/macrocyclic-peptide inhibitors. Most notably, in vivo CRISPR base-editing of PCSK9 (e.g., VERVE-102), which produced dose-dependent reductions in PCSK9 and LDL-C of up to 88% and 62%, respectively, in an early-phase trial, raising the prospect of a single-dose, durable therapeutic [26].

### 2.2. Structural Genetics and Clinical Implications of Pcsk9 Variants

In the regulation of LDL-C homeostasis, PCSK9 has become recognized as a mechanistically distinct element that connects molecular structure to clinical outcomes [5]. Naturally occurring GOF and LOF variants in PCSK9 respectively alter the pathway toward either pronounced monogenic hypercholesterolemia or cardioprotective effects (Figure 1C), offering robust human genetic evidence supporting both causality and therapeutic potential [10,13,27]. GOF and LOF variants produce reciprocal phenotypes, autosomal dominant hypercholesterolemia versus cardioprotective hypocholesterolemia. The structural and mechanistic basis of this divergence is examined in detail in Section 3. These results position PCSK9 as a leading example of how “human knockout” biology can contribute to advancements in drug development. Individuals who naturally carry LOF alleles and remain healthy provide in vivo proof that PCSK9 is a safe and efficacious pharmacological target, a logic that directly underpinned the clinical development of PCSK9-based therapies [28,29]. Alongside these therapeutic applications, well-documented monogenic examples such as the S127R GOF variant remain highly valuable for diagnostic purposes, cascade screening, and mechanistic interpretations of LDLR pathway dysfunction [30].

**Figure 2:**
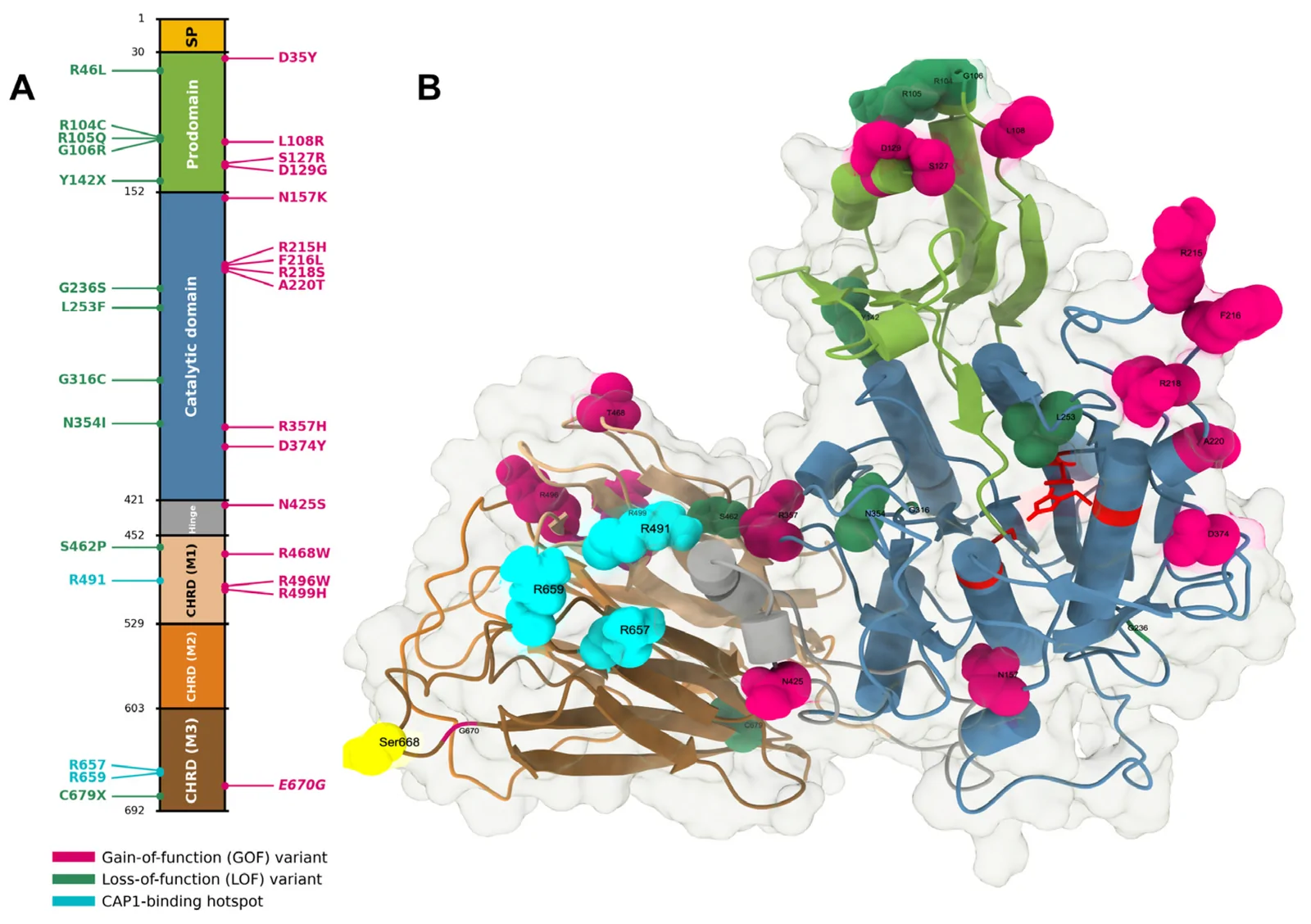
Structural domains and clinical variant landscape of PCSK9. (**A**) Linear domain map (residues 1–692, pre-pro-hPCSK9 numbering) annotated with all 27 variants curated in Table 1: gain-of-function (GOF) variants in pink/magenta and loss-of-function (LOF) variants in green. (**B**) The same GOF/LOF variant positions mapped onto a 3D structural composite of PCSK9 in its closed conformation, using matched domain and variant colors: prodomain (green, residues 61–152) is from the crystal structure of the PCSK9 CHRD/P1.40 complex (PDB 3P5C); catalytic domain (blue, 153–421), hinge (gray, 422–452), and C-terminal CHRD (453–692, subdivided into M1 tan, M2 orange, and M3 brown) are from a MODELLER v10.8 homology model cross-validated against an independent AlphaFold prediction (see Figure 3 legend for full modeling provenance). The catalytic triad (Asp186, His226, Ser386) is shown in red, the FAM20C phosphoacceptor Ser668 in yellow, and the CAP1-binding hotspots (Arg491, Arg657, Arg659) in cyan. Twenty-five of the 27 variants are mapped in (**B**) as labeled Cα spheres (L108R, S127R, D129G, N157K, R215H, F216L, R218S, A220T, R357H, D374Y, N425S, R468W, R496W, R499H, E670G, R104C, R105Q, G106R, Y142X, G236S, L253F, G316C, N354I, S462P, C679X); D35Y and R46L fall outside the modeled range (61–692) and are shown only in (**A**).

This tight genotype–mechanism–phenotype relationship motivates structural–genetic analysis. Mapping variants onto PCSK9’s domain architecture and 3D interaction surfaces (Figure 2) helps explain why particular substitutions alter LDLR affinity, pH-dependent conformational behavior, or intracellular processing. Current research demonstrates that variants influencing the pro domain–catalytic domain configuration or stabilizing surfaces within the PCSK9–LDLR complex can intensify receptor degradation independently of PCSK9’s protease activity, underscoring the productive synergy between structural biology and genetics for elucidating PCSK9 function [31,32].

**Figure 3:**
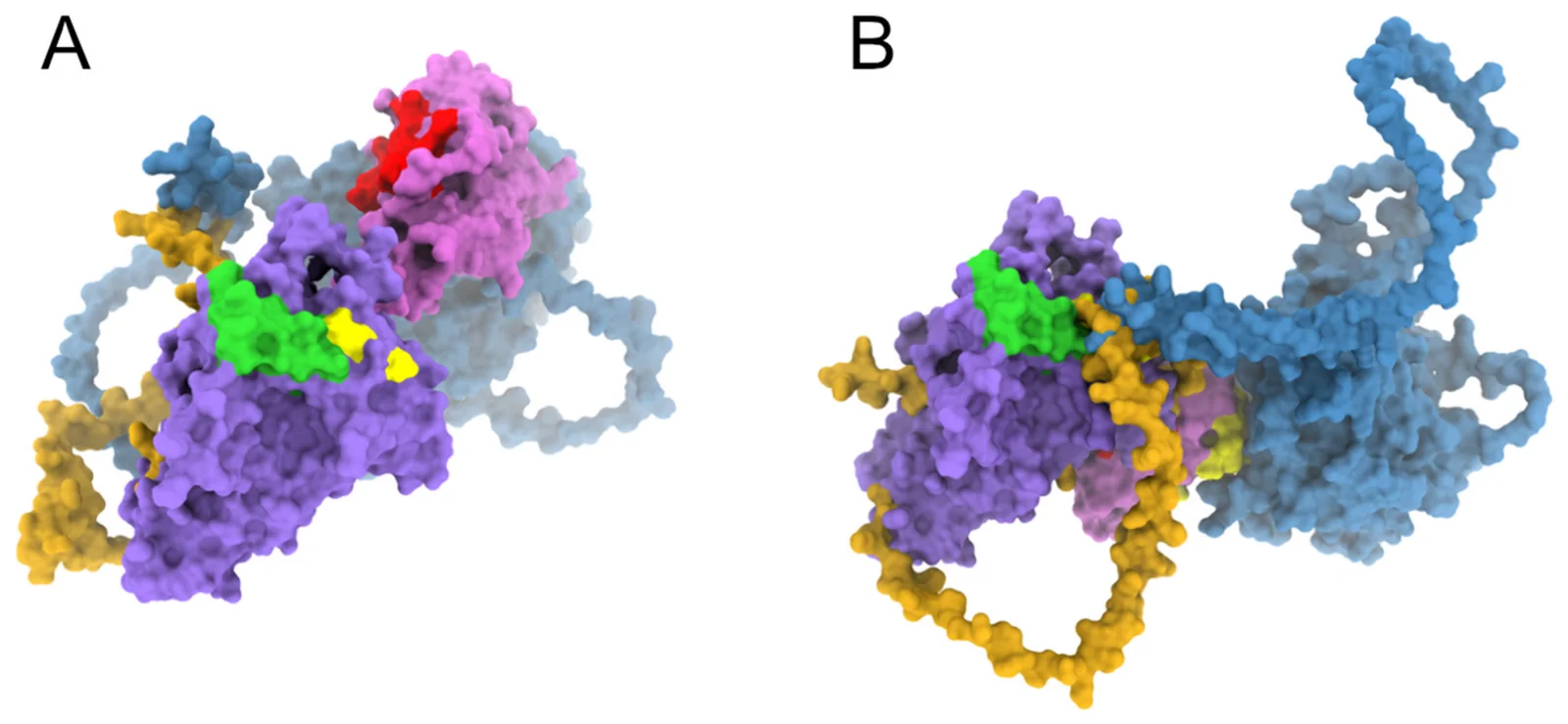
The PCSK9-FAM20C-CAP1 ternary complex in open and closed CHRD conformations, with the FAM20C-, CAP1-, and LDL receptor (LDLR)-binding footprints mapped onto the PCSK9 surface. This composite is discussed in detail in Section 3.3 (E670G case study). (**A**) Open conformation (left) and (**B**) closed conformation (right) of the PCSK9-FAM20C-CAP1 ternary assembly, shown side by side after rigid-body superposition of both models onto the same fixed PCSK9 core anchor (chain A, residues 61–452; RMSD 0.0004 Å over 392 shared Cα pairs). PCSK9 is rendered as a two-toned solid surface, core (residues 61–452) in medium purple and CHRD (453–692) in orchid, with the hinge backbone (422–452) additionally traced as a thin indigo tube. In (**A**), FAM20C engages the Ser668-Xaa-Glu670 motif (CHRD M3). FAM20C (steelblue) and CAP1 (goldenrod) are shown as solid surfaces alongside PCSK9 in both panels, with no atomic detail, to depict the ternary complex without implying resolved side-chain contacts for these AlphaFold/HADDOCK-derived docking models. Three residue footprints are mapped onto PCSK9’s surface: yellow for the FAM20C-contact footprint (Table S1, HADDOCK2.4 model), red for the CAP1-contact footprint (Table S2, AlphaFold2-multimer model), and lime green for the LDL receptor (LDLR) EGF-A-domain binding site (catalytic-domain residues 153–155, Arg194, and 367–381, including the Asp374 gain-of-function hotspot)—unlike the other two footprints, this one is taken directly from the experimentally determined PCSK9:EGF-A crystal structure ([33], PDB 3BPS) rather than a computational model; LDLR itself is not shown, as it is not part of the complex depicted here, and this footprint is included only for spatial reference relative to the FAM20C/CAP1 sites. In (**B**), CAP1 clashes directly with the catalytic domain surface (94 atom pairs <1.3 Å), showing that the CHRD M1/M3 surface it requires is occluded until PCSK9 adopts the open conformation shown in (**A**). FAM20C and CAP1 occupy overlapping CHRD M3 footprints (Tables S1 and S2), indicating sequential rather than simultaneous engagement, consistent with the reported enhancement of CAP1 binding by Ser668 phosphorylation. See Figure 2 for the PCSK9 domain architecture and the full clinical variant landscape (Table 1) mapped onto this same structural composite.

Translating these structural insights into clinical practice has profoundly reshaped the therapeutic landscape for hypercholesterolemia. Because LDLR degradation is the shared endpoint of PCSK9 activity, therapies that block this axis endpoint exploit the same underlying biological mechanism, whether by extracellular neutralization of circulating PCSK9 with monoclonal antibodies or by hepatic silencing of PCSK9 production with siRNA [34]. Accordingly, emerging therapies such as ezetimibe and PCSK9 inhibitors have been integrated into current lipid-lowering strategies to facilitate more substantial LDL-C reductions and enhance cardiovascular outcomes, exemplifying a progressive, mechanism-driven approach to tackling the global impact of hypercholesterolemia [35] (Figure 1D). While monoclonal antibodies and siRNA therapeutics require injection, an oral alternative is now approved. The macrocyclic peptide inhibitor enlicitide decanoate (Lipfendra) demonstrated 56–59% LDL-C reductions versus placebo in the pivotal CORALreef Lipids and CORALreef HeFH Phase 3 trials and received FDA approval on 16 July 2026 as the first oral PCSK9 inhibitor [36] (Section 4.4), representing a progressive evolution in targeting this pathway; a third trial, the CORALreef Outcomes cardiovascular-outcomes study, remains ongoing.

**Table 1:** Major PCSK9 gain- and loss-of-function variants: domain location, mechanistic category, and associated clinical phenotypes.

Variant	Domain	Type	Mechanistic Category	Molecular Effect	LDL-C Effect	Clinical Phenotype	Reference
S127R	Pro domain	GOF	Trafficking/intracellular processing	Increased LDLR degradation; enhanced trafficking	↑↑ LDL-C	Severe familial hypercholesterolemia	[30,37]
D374Y	Catalytic domain	GOF	Increased LDLR binding affinity	Stronger binding to LDLR; stabilized complex	↑↑ LDL-C	Severe early-onset ASCVD	[32,37,38]
F216L	Catalytic domain	GOF	Protein stability/activity	Increased PCSK9 activity	↑ LDL-C	Familial hypercholesterolemia	[32,39]
R496W	CHRD	GOF	Trafficking/receptor degradation	Enhanced receptor degradation	↑ LDL-C	Autosomal dominant hypercholesterolemia	[32,39]
R46L	Pro domain	LOF	Reduced LDLR binding/stability	Reduced affinity for LDLR	↓ LDL-C	Cardioprotection	[40,41]
Y142X	Pro domain	LOF	Truncation/secretion defect	Truncated protein; reduced secretion	↓↓ LDL-C	Markedly reduced CHD risk	[41,42]
C679X	CHRD	LOF	Truncation/loss of function	Loss of functional PCSK9	↓↓ LDL-C	Strong cardioprotective phenotype	[42,43]
E670G	CHRD (M3)	GOF (mild)	Disrupted Fam20C phosphorylation/CAP1 recruitment	Loss of S-X-E phosphomotif (Glu670); reduced CAP1 binding in vitro	↑ LDL-C (modest)	Mild hypercholesterolemia; increased CAD risk (East Asian cohorts)	[44,45]
D35Y	Pro domain	GOF	Prodomain regulatory function	Altered lipoprotein particle composition; reduced CETP-mediated HDL-C efflux	↑ LDL-C	Autosomal dominant hypercholesterolemia (French families)	[12,46,47]
L108R	Pro domain	GOF	Prodomain regulatory function	Altered lipoprotein particle composition; reduced CETP-mediated HDL-C efflux	↑ LDL-C	Autosomal dominant hypercholesterolemia (French families)	[12,46,47]
D129G	Pro domain	GOF	Trafficking/intracellular processing	Missense substitution grouped with established GOF S127R	↑ LDL-C	Familial hypercholesterolemia (New Zealand cohort)	[12,46,48]
N157K	Pro domain	GOF	Trafficking/intracellular processing	Substitution associated with elevated PCSK9 activity	↑ LDL-C	Hypercholesterolemia	[12,46]
R215H	Catalytic domain	GOF	Protein stability/activity	Increased PCSK9 activity	↑ LDL-C	Hypercholesterolemia	[12,46,49]
R218S	Catalytic domain	GOF	Protein stability/activity	Missense substitution near the furin-cleavage region	↑ LDL-C	Autosomal dominant hypercholesterolemia (French cohort)	[12,46,50,51]
A220T	Catalytic domain	GOF	Trafficking/receptor degradation	Enhanced receptor degradation	↑↑ LDL-C	Homozygous-pattern familial hypercholesterolemia (pediatric, Dutch cohort)	[12,46,52]
R357H	Catalytic domain	GOF	Increased LDLR binding affinity	Stronger binding to LDLR	↑ LDL-C	Autosomal dominant hypercholesterolemia (French cohort)	[12,46,51]
N425S	Hinge	GOF	Trafficking/receptor degradation	Identified alongside R496W in a homozygous FH patient with mutated LDLR	↑↑ LDL-C	Homozygous familial hypercholesterolemia phenotype	[12,46,53]
R468W	CHRD	GOF	Trafficking/receptor degradation	Enhanced receptor degradation	↑ LDL-C	Autosomal dominant hypercholesterolemia (French cohort)	[12,46,51]
R499H	CHRD	GOF	Trafficking/receptor degradation	Decreased LDLR expression in vitro	↑ LDL-C	Familial hypercholesterolemia (Spain, Italy)	[12,46,54]
R104C	Pro domain	LOF	Reduced LDLR binding/stability	Improved LDL-C clearance rate (double mutant with V114A)	↓ LDL-C	Reduced LDL-C	[12,46,55]
R105Q	Pro domain	LOF	Reduced LDLR binding/stability	Lower total cholesterol in carriers	↓ LDL-C	Reduced cholesterol (Pakistani FH families)	[12,46,56]
G106R	Pro domain	LOF	Reduced LDLR binding/stability	Identified in hypocholesterolemia population screening	↓ LDL-C	Hypocholesterolemia	[12,46,57]
G236S	Catalytic domain	LOF	ER retention/secretion defect	Prevents PCSK9 release from the endoplasmic reticulum	↓↓ LDL-C	Hypocholesterolemia	[12,46,49]
L253F	Catalytic domain	LOF	Reduced LDLR binding/stability	Correlated with diminished plasma LDL-C	↓ LDL-C	Reduced LDL-C	[12,46,58]
G316C	Catalytic domain	LOF	Reduced LDLR binding/stability	Associated with hypocholesterolemia and hepatic steatosis	↓ LDL-C	Hypocholesterolemia; hepatic steatosis	[12,46,59]
N354I	Catalytic domain	LOF	Autocatalytic processing defect	Failure of autocatalytic cleavage; ER retention	↓↓ LDL-C	Hypocholesterolemia	[12,46,49]
S462P	CHRD	LOF	ER retention/secretion defect	Prevents normal C-terminal domain folding; blocks ER release	↓↓ LDL-C	Hypocholesterolemia	[12,46,60]

LDL-C effect symbols, the E670G classification rationale, and Table 1 citation-sourcing notes are provided in the Supplementary Materials (Note S1). LDL-C Effect key: ↑↑/↓↓ denote large (monogenic-scale) increases/decreases in plasma LDL-C; ↑/↓ denote moderate effects.

#### Authors’ Preliminary Interaction Analyses: The Pcsk9-Fam20c-Cap1 Ternary Complex

To translate this structural-genetic framework into a concrete structural hypothesis for the CHRD regulatory axis (see also Section 2.4.5), we constructed a representative PCSK9-FAM20C-CAP1 ternary composite (Figure 3; Supplementary Materials). Because no experimental structure of this assembly exists, each partner was modeled independently before assembly: the FAM20C kinase domain was AlphaFold-predicted and validated against the crystal structure of its Caenorhabditis elegans ortholog (PDB 4KQA, 0.94 Å Cα RMSD over the conserved kinase-domain core), and the CAP1 SH3-binding linker (residues 214–337) was predicted with AlphaFold2-Multimer/ColabFold following the approach of Fruchart-Gaillard et al. [61]. These independently predicted components were then assembled onto the PCSK9 CHRD using HADDOCK2.4, anchored on the Ser668 phosphoacceptor and its catalytically analogous FAM20C active-site residue, yielding the interface maps summarized in Tables S1 and S2. Without using any mutagenesis data as a docking constraint, the resulting CAP1 interface footprint reproduces the CAP1-binding hotspot triad (Arg491, Arg657, Arg659) independently identified by alanine scanning and bio-layer interferometry [61], and places FAM20C and CAP1 on overlapping CHRD M3 surfaces that are occluded in the closed PCSK9 conformation, consistent with the sequential, phosphorylation-gated engagement model proposed for this axis (Section 3.3).

Several disease-associated variants map directly onto these computationally defined interfaces. E670G disrupts the Ser668-Xaa-Glu670 FAM20C recognition motif itself (Section 3.3; Table S1), and within CHRD M1, the loss-of-function variant S462P falls directly within, and the gain-of-function variant R468W lies immediately adjacent to, the AlphaFold2-Multimer-predicted CAP1 contact surface (Table S2, residues 458–481). These positions offer concrete, testable hypotheses for how individual variants might perturb FAM20C- or CAP1-binding independently of LDLR engagement, illustrating how this structural framework could be applied prospectively to other CHRD variants as they are reported, rather than requiring a bespoke computational analysis for each one.

We emphasize the preliminary nature of this composite. As detailed in the Supplementary Materials, it is a rigid-body docking assembly that has not been subjected to energy minimization or molecular dynamics refinement, the phosphorylated Ser668 state was approximated by its unphosphorylated docking-active-site proxy rather than modeled explicitly, and the open-CHRD conformation required for FAM20C/CAP1 access was imposed by manual rigid-body rotation about the hinge rather than allowed to emerge dynamically. Validating and extending this preliminary model with physics-based simulation is therefore a natural next step, discussed in Section 5.3.

### 2.3. Bioinformatic Approaches to Understanding Pcsk9 Variants

#### Literature Findings: Structure- and Dynamics-Based Characterization of GOF and LOF Variants

Static structure-prediction tools such as AlphaFold and Rosetta have made high-quality structural models of PCSK9 and its variants increasingly accessible, complementing the experimental crystal structures already available for this target (Section 2.1). However, these methods generally return a single, time-averaged conformation and cannot, on their own, capture the conformational dynamics that govern protein function. Molecular dynamics (MD) simulations address this gap directly: by propagating a structure through time under an explicit or implicit physical force field, MD samples the ensemble of conformations a protein actually populates in solution rather than a single static snapshot. This distinction matters functionally for PCSK9, because both loss-of-function (LOF) and gain-of-function (GOF) variants frequently act not by abolishing a static binding surface but by shifting the underlying conformational equilibrium of flexible regions, including the hinge-CHRD interface and the intrinsically disordered CHRD M3 loop, in ways that a single predicted structure cannot reveal. The following subsections summarize how MD-based and related enhanced-sampling approaches have been applied to characterize these dynamics in the published literature, present our own preliminary structural analysis of the PCSK9-FAM20C-CAP1 interaction network, and outline priority directions for extending this work (Section 5.3).

Bioinformatic workflows have become essential tools for interpreting PCSK9 sequence variability, enabling researchers to systematically link genotype, structure, and LDLR-binding phenotypes (summarized in Table 2). At the variant level, pathogenicity assessment typically begins with in silico predictors, such as conservation-based, structure-based, and ensemble-based scores, to prioritize missense candidates for subsequent functional validation [62]. This strategy is reflected in recent analyses of PCSK9 variants that integrate multiple stability and function predictors to efficiently identify potentially deleterious substitutions [63]. To enhance predictive accuracy beyond static scoring methods, molecular dynamics (MD) simulations are increasingly employed to evaluate how specific substitutions modify the PCSK9–LDLR interface. These approaches include long-timescale simulations comparing wild-type PCSK9 with clinically observed variants (such as the gain-of-function substitutions D374Y and S127R) and engineered interface mutants E498A and R499G. These simulations are followed by MM/GBSA binding energy calculations to directly associate altered conformational dynamics with predicted changes in LDLR affinity [64]. Independently, residue-interaction-network and MM/PBSA analysis of the gain-of-function substitutions S127R and D374Y showed both confer markedly increased LDLR-binding affinity relative to wild-type, via distinct interfaces (prodomain-beta-propeller for S127R; catalytic-domain-EGF(A) for D374Y), providing an early mechanistic rationale for interface-disruptive small-molecule strategies [65]. More recently, AlphaFold-based modeling of full-length PCSK9 before and after propeptide cleavage found that the mature N-terminal region undergoes a loop-to-helix transition upon cleavage, illustrating how static AlphaFold models can reveal cleavage-dependent conformational states that complement the MD-based dynamics discussed throughout this section [66].

Beyond the individual gain-of-function variants highlighted above, molecular dynamics has increasingly been applied across a much wider swath of the PCSK9 variant spectrum. On the loss-of-function side, independent simulation studies of PCSK9-LDLR complexes carrying R93C, N298D, and A511V, and of a separate loss-of-function panel using the GROMACS/Gromos53a6 force field, both report increased flexibility and reduced complex stability relative to wild-type, consistent with the mechanistic picture established for the loss-of-function substitutions discussed above [67,68]. Population-specific variant sets have received comparable treatment: Perturb Response Scanning and principal component analysis of the Brazilian cohort variants rs505151 (corresponding to the E670G polymorphism discussed above) and rs562556 revealed altered dynamical coupling between the prodomain and the CHRD [69], while a Vietnamese familial hypercholesterolemia registry study combined structural modeling with MD to characterize how the Leu23_Gly24insLeu insertion perturbs the PCSK9-APOB-LDLR complex [70]. Complementary force-field comparisons and peptide-binding studies have further probed the gain-of-function interface: an effective-polarizable-bond force field better reproduced binding-affinity trends across four PCSK9-EGF(A) gain-of-function mutants than standard AMBER parameters [71], microsecond-scale MD paired with HEK293FT functional assays linked p.(E32K) and p.(R469W) to increased backbone fluctuation and altered hydrogen-bonding at the prodomain-CHRD interface [72], and extended MD and metadynamics studies of the D374Y substitution characterized how it alters binding of food-derived lupin peptide inhibitors at the PCSK9-LDLR interface [73,74]. Finally, AlphaFold-based modeling has begun to extend this variant-level structural characterization beyond the classical GOF/LOF panel: p.(R160Q), identified incidentally in a hypercholesterolemic subject, is predicted to displace the P’ helix (residues 153–168) away from the LDLR-binding catalytic domain [75], and a C-terminal frameshift variant, p.(Val675Leufs*130), is predicted to disrupt the CHRD’s terminal β-sheet entirely [76]. Taken together, this broader body of work indicates that MD and AI-based structural modeling have already been applied across a substantially wider range of PCSK9 variants and interaction partners than the individual case studies above capture in isolation, reinforcing the case for systematic, structure-based variant characterization made throughout this review.

Classical computer-aided drug design (CADD), structure- and ligand-based virtual screening, pharmacophore modeling, molecular docking, and quantitative structure–activity relationship (QSAR) analysis remain the entry point for PCSK9 inhibitor discovery. Altogether they provide the reference compounds against which newer AI-driven methods are benchmarked. Docking algorithms generate and rank candidate binding poses using scoring functions that fall into three broad classes. First, physics-based functions (e.g., the semi-empirical force fields underlying AutoDock and GoldScore) that approximate binding free energy from van der Waals, electrostatic, and desolvation terms. Second, empirical functions that regress a weighted sum of interaction descriptors against experimental affinity data. Third, knowledge-based statistical potentials derived from atom-pair contact frequencies observed across solved protein–ligand structures, with machine-learning-based scoring now emerging as a fourth class trained directly on large complex datasets [77,78]. This classical pipeline has been applied directly to PCSK9. Iqbal et al. combined pharmacophore-based virtual screening with in silico docking and in vitro validation to identify brazilin, a homoisoflavonoid from Caesalpinia sappan, as a PCSK9 inhibitor [79]. Ten pharmacophore models were built and validated in LigandScout against DUD-E-derived active/decoy sets (best model AUC  =  0.93, enrichment factor at 1%  =  34.0). These models were then used to filter eleven natural compounds from the HerbalDB library by Lipinski’s rule-of-five. Finally, the candidates were docked using AutoDock Vina in PyRx against a 1.53 Å PCSK9 crystal structure (PDB 6U26), with the binding site defined by Asp360, Cys358, Arg357, Arg476, Arg458, and Pro331. Among screened candidates, Brazilin achieved the most favorable predicted binding affinity (−9.0 to −9.1 kcal/mol), superior to the cocrystallized reference ligand (−8.4 kcal/mol). The complex was stabilized by seven hydrogen bonds together with π-alkyl and π-sigma contacts. This computational ranking was subsequently confirmed experimentally through a CircuLex PCSK9–LDLR binding assay, yielding an IC50 of 2.19 μM, more potent than the crude C. sappan extract (IC50 0.72 μg/mL) and several previously reported small-molecule PCSK9 inhibitors. Notably, brazilin subsequently reappears as the positive-control benchmark against which the generative, pharmacophore-guided AI design pipeline described later in this section evaluated its lead candidate [80], illustrating how classical virtual screening campaigns continue to define the activity thresholds and reference chemotypes that calibrate next-generation, AI-driven PCSK9 inhibitor design.

Building on this classical docking foundation, more integrated pipelines combine structure-based virtual screening with MD refinement to facilitate the identification and optimization of small-molecule binders targeting PCSK9. Here, molecular docking serves as an initial filter for compound libraries, MD assesses pose stability, and free-energy calculations coupled with hotspot mapping inform residue prioritization for medicinal chemistry optimization [63].

Empirical scoring methods, such as FoldX’s additive, knowledge-derived free-energy terms [81], are complemented by end-point free-energy methods, such as MM-PBSA and MM-GBSA. These methods extract quantitative binding free energies directly from MD trajectories and are increasingly applied to dissect how individual PCSK9 variants perturb the LDLR interface rather than merely describing whether binding occurs. As a representative example, GROMACS-based MM/GBSA analysis (calculated with the gmx_MMPBSA tool, which implements the Poisson–Boltzmann and generalized Born surface-area methods, respectively, atop the chosen molecular-mechanics force field) of triplicate 500-ns trajectories for wild-type, E498A, and R499G LDLR–PCSK9 complexes revealed markedly different predicted binding free energies (−33.07, −63.81, and −24.21 kcal/mol, respectively). The gain-of-function-like E498A substitution nearly doubled the wild-type binding affinity, while R499G weakened it, differences attributable to divergent conformational sampling of the LDLR β-propeller domain rather than to local contacts alone [64]. Identifying which specific residues drive this affinity difference is itself a computational goal. Computational alanine scanning systematically mutates each interface residue to alanine in silico to evaluate its individual contribution to the binding free energy. When paired with interaction-entropy-corrected MM/GBSA, this method accurately maps PCSK9–LDLR hot-spot residues to guide inhibitor design [82]. Ultimately, such end-point free-energy decomposition offers a route to rank both existing familial hypercholesterolemia-associated (FH-associated) variants and candidate small-molecule or peptide inhibitors on a common quantitative energetic scale, rather than relying on docking scores or MD stability alone.

A complementary strategy targets not the bound complex but the intrinsic thermodynamic stability of PCSK9 itself. This is achieved using ΔΔG (ddG) predictors such as FoldX [81] and Rosetta’s [83] ddg_monomer or cartesian_ddg protocols, which estimate the change in protein folding free energy caused by a point substitution from a single structure. Cross-method benchmarking in the CAGI5 blind stability challenge found that FoldX, Rosetta, and the sequence-based DDGun3D [84] achieved the strongest correlation with experimentally measured mutational effects, although all methods remain comparatively poor at anticipating destabilizing loss-of-function substitutions and are sensitive to whether the input structure is an experimental structure or a homology model [85]. Applied qualitatively to PCSK9, whole-exome sequencing and computational structural analysis of a rare gain-of-function variant (R496W) identified in a Saudi hypercholesterolemia family predicted that the substitution destabilizes the native fold and alters secondary and tertiary structure. Simultaneously, it impairs binding to both LDLR and the therapeutic antibody alirocumab, illustrating how stability prediction can complement sequence-based variant-effect classifiers such as AlphaMissense [86] by distinguishing folding defects from interface defects [31]. Systematic ddG scanning across the full spectrum of reported PCSK9 missense variants, rather than isolated case studies, remains a largely open computational opportunity for this target, one that could yield a comprehensive stability map linking every reported PCSK9 variant to its predicted structural and clinical consequence.

#### Allosteric Networks and Cryptic-Pocket Discovery via Molecular Dynamics

Unlike a deep enzymatic pocket, the canonical PCSK9–LDLR interface is a broad, comparatively flat protein–protein surface. Because this topology is intrinsically difficult to target directly with small molecules, researchers are actively seeking alternative, indirect binding sites. To map these remote targets, several groups have turned to dynamical network analysis [87]. This method builds a residue–residue correlation or community network from MD trajectory ensembles to trace how a local perturbation propagates to a distal functional site. It offers a route to identifying indirect, allosteric strategies for restoring LDLR function, rather than competing head-on for the difficult orthosteric interface. Applying this logic directly to PCSK9, correlation analysis comparing LDLR-bound and unbound trajectories identified a distal loop region whose conformational state correlates bidirectionally with LDLR-binding affinity. Variants or ligands that rigidify or displace this loop propagate allosterically to the binding interface, establishing a mechanistic basis for modulating the PCSK9–LDLR protein–protein interaction from a site distinct from the interface itself [88]. Such analyses are implemented within dedicated dynamical-network toolkits of standard MD engines such GROMACS [89] correlationplus module. These toolkits compute generalized correlation coefficients and shortest-path betweenness from trajectory ensembles to flag residues that are structurally distant but dynamically coupled [87]. For PCSK9, this reframes the search for gain-of-function-reversing or LDLR-restituting interventions away from the crowded, shallow orthosteric interface and toward dynamically coupled but topologically distinct regions of the protein.

Extending this allosteric logic into structure-based drug discovery, mixed-solvent molecular dynamics (MSMD) has been used to search PCSK9 for druggable sites beyond the canonical LDLR-binding face. In MSMD, small organic probe molecules such as isopropanol, acetonitrile, or benzene are co-simulated with the protein in explicit solvent to map transient, cryptic binding pockets that are invisible in any single static structure. Using six chemically diverse probes, MSMD simulations of the PCSK9 C-terminal domain (CTD) revealed a previously uncharacterized cryptic allosteric pocket approximately [90] Å from the LDLR-binding interface. The opening of this pocket was driven by disruption of the R580–E612 salt bridge. Furthermore, network-based correlation analysis linked this CTD pocket to the LDLR interface through relay residues, including H574 and V610, providing a concrete, druggable target for allosteric PCSK9 inhibitors that would not compete directly with LDLR or with catalytic-domain-directed antibodies [91]. Because this pocket lies far from both the docking site characterized for brazilin (discussed earlier) [79], it illustrates how cryptic-pocket mapping can expand the druggable surface of PCSK9 beyond the sites explored by classical virtual screening and antibody engineering.

Complementing pure MSMD-based detection, hybrid pipelines such as CrypToth pair mixed-solvent probe simulations with topological data analysis, specifically persistent homology. This approach ranks candidate cryptic-pocket hotspots by the persistence and volume of the transient cavities they form, outperforming machine learning- or chemoinformatics-based cryptic-pocket predictors on benchmark sets of experimentally confirmed cryptic sites [92]. Applying this topological ranking step to the PCSK9 CTD probe trajectories described above could help prioritize which of several candidate sub-pockets within the R580–E612 region is most tractable for medicinal chemistry follow-up.

**Table 2:** Computational methods applied to PCSK9 structure, dynamics, and inhibitor design, organized by method category, representative tools, PCSK9-specific application, and key finding.

Method Category	Representative Tools	PCSK9 Application	Key Finding	Ref.
Variant-effect prediction	AlphaMissense; ESM1b	Genome-scale missense pathogenicity scoring	Reaches about 90% AUC on ClinVar-derived benchmarks; neither tool has yet been benchmarked against the curated PCSK9 variant set	[86,93]
ΔΔG stability prediction	FoldX; Rosetta ddg_monomer/cartesian_ddg; DDGun3D	Predicting fold-destabilizing effects of point substitutions	Strongest methods correlate well with experimental stability data in CAGI5 benchmarking, but remain weak at flagging destabilizing LOF variants	[81,83,84,85]
Molecular dynamics and free-energy decomposition	GROMACS/AMBER (ff14SB, CHARMM36m); MM-GBSA; MM-PBSA	Wild-type versus E498A/R499G LDLR–PCSK9 complexes	E498A binding free energy nearly doubled the wild-type value; R499G weakened it	[64,94,95]
Computational alanine scanning	Interaction-entropy-corrected MM/GBSA	Mapping PCSK9–LDLR hot-spot residues	Identifies residues that drive affinity differences to guide inhibitor design	[82]
Dynamical-network and allosteric-coupling analysis	correlationplus; generalized correlation and betweenness mapping	Distal-to-interface allosteric communication	Located a distal loop whose conformational state correlates with LDLR-binding affinity	[87,88,89]
Mixed-solvent MD and cryptic-pocket mapping	MSMD probe simulations; CrypToth (persistent homology)	C-terminal domain cryptic-pocket discovery	Revealed a druggable pocket about [90] Å from the LDLR interface, gated by the R580–E612 salt bridge	[91,92]
Classical docking and CADD	AutoDock Vina; PyRx; pharmacophore modeling (LigandScout)	Virtual screening for small-molecule inhibitors	Identified brazilin as a PCSK9 inhibitor, later confirmed in a CircuLex binding assay	[77,78,79]
AI-driven structure prediction	AlphaFold2; AlphaFold-Multimer; AlphaFold3	PCSK9 monomer and complex modeling	Confidently predicts the ordered core, but confidence drops sharply across the flexible CHRD tail	[90,96,97,98]
Generative protein and antibody design	RFdiffusion; ProteinMPNN; RFantibody; OptMAVEn	De novo nanobody and miniprotein binder design	Validated experimentally across multiple antigens, though not yet applied directly to PCSK9	[99,100,101,102,103,104]
Sequence-based peptide design	Protein-language-model peptide generation; peptide-PROTAC engineering	Peptide binder and degrader design	A computationally designed 10-mer peptide-PROTAC degraded PCSK9 and lowered LDL-C in vivo	[105,106]

MD, molecular dynamics; MM-GBSA/MM-PBSA, molecular mechanics generalized Born/Poisson–Boltzmann surface area; CADD, computer-aided drug design; AUC, area under the receiver operating characteristic curve. Reference numbers correspond to the numbered reference list in the References section.

### 2.4. Integrative Computational and Machine-Learning Frontiers

#### 2.4.1. Antibody and Biologic Engineering

Turning from small-molecule pocket discovery to biologics, antibody engineering represents a complementary computational frontier for PCSK9 inhibition. The clinically dominant PCSK9-targeted therapeutics, the monoclonal antibodies evolocumab and alirocumab, act by sterically blocking the PCSK9–LDLR interaction. The optimization of these and next-generation biologics increasingly relies on computational epitope mapping and structure-guided affinity maturation rather than affinity screening alone. Camelid-derived single-domain antibodies raised against a linear epitope in the PCSK9 hinge domain have been structurally characterized to define the minimal binding determinants required for LDLR-blocking activity, offering a smaller, more stable scaffold than full-length IgGs for further computational engineering [99]. More broadly, frameworks such as OptMAVEn simulate in silico the steps of antibody variable-region generation and affinity maturation against a defined antigen epitope, combinatorially assembling germline-derived framework and CDR fragments and scoring candidate paratopes for shape and electrostatic complementarity to the target epitope, in principle enabling epitope-directed design of next-generation anti-PCSK9 biologics ahead of wet-lab screening [100].

Pushing this boundary further, the David Baker laboratory (Institute for Protein Design) has released a connected suite of open tools spanning this entire design cycle. This includes RoseTTAFold2 for structure prediction [101], ProteinMPNN for sequence design given a backbone [102], and RFdiffusion for de novo backbone generation.

Building on this architecture, a specialized structure-conditioned extension known as RFantibody fine-tunes the model specifically for antibody CDR-loop design. It generates atomically accurate, epitope-targeted heavy- and light-chain loops de novo rather than combinatorially assembling germline fragments, and has been validated experimentally across multiple target antigens [103]. Coupling such epitope-directed design frameworks with the AlphaFold-Multimer-based complex prediction already used to model PCSK9 assemblies [90] represents a natural extension for computationally engineering biologics against epitopes, such as the CTD allosteric pocket described above, that remain inaccessible to the current generation of catalytic-domain-directed antibodies.

#### 2.4.2. Ai-Driven Variant Pathogenicity

Two structure-aware deep-learning predictors now dominate genome-scale missense pathogenicity assessment and are directly applicable to the PCSK9 variant catalogue in Table 1. AlphaMissense repurposes AlphaFold’s structure module and evolutionary-density training to classify every possible human missense substitution as likely benign, likely pathogenic, or ambiguous, reaching approximately 90% AUC against curated ClinVar-derived benchmarks without being trained on clinical labels directly [86]. Alternatively, ESM1b, a 650-million-parameter protein language model, instead scores variant effects purely from evolutionary sequence context, without requiring an explicit 3D structure, and has been benchmarked genome-wide against clinical variant sets [93]. To our knowledge, neither tool has yet been systematically benchmarked against the curated PCSK9 GOF/LOF/polymorphism set summarized in Table 1, despite both being freely available as precomputed genome-wide predictions. Doing so would provide an immediate, low-cost complement to the structure-based and MD-based pathogenicity workflows described above and represents a concrete, near-term extension of this review’s framework.

#### 2.4.3. Methodological Heterogeneity in Md Simulations

Published PCSK9-focused simulations illustrate this methodological diversity well, differing substantially in their technical implementation. For example, Azhar et al. modeled the LDLR–PCSK9 complex (PDB 3P5C) and its E498A/R499G variants using GROMACS with the CHARMM36m force field, running triplicate 500-ns simulations per system (310.15 K, NPT ensemble, 2-fs timestep, 3.5 μs aggregate sampling), Their MM/GBSA analysis identified E498A as markedly more stable (−63.81 kcal/mol) than wild-type (−33.07 kcal/mol) or R499G (−24.21 kcal/mol) [64]. By contrast, a parallel virtual-screening study combined Schrödinger (HTVS/SP/XP docking, LigPrep, QikProp) and AutoDock Vina screening of a 200,888-compound library against the PCSK9–LDLR EGF-A interface (PDB 3BPS). This was paired with AMBER [29] simulations (ff14SB protein force field, GAFF/AM1-BCC ligand parameters, TIP3P water, Langevin thermostat, triplicate 200-ns production runs) and a free-energy calculation protocol that adds an interaction-entropy correction to MM/GBSA and, for selected systems, replaces fixed-charge AMBER electrostatics with polarized protein-specific charges (PPC) derived from QM/MFCC fragmentation. Per-residue alanine-scanning and dynamic cross-correlation matrix (DCCM) analysis further mapped allosteric coupling across the complex [82]. Complementary ligand-based strategies illustrate the same lack of a single standard workflow. Pharmacophore models have been built from known PCSK9–LDLR disruptors and screened against the ZINC database [107], and, most recently, a hybrid pipeline pairing pharmacophore-filtered virtual screening of 4495 terpenoid compounds with AI-guided fragment substitution, a LogitBoost bioactivity classifier (AUC = 0.864), quantum-chemical refinement, and 200-ns MD with free-energy validation [80]. Taken together, these studies demonstrate that structure-based and AI-augmented ligand design are converging within the PCSK9 field. However, they also reveal that force field choice (AMBER [94] vs. CHARMM [95]), water models, ensembles, aggregate sampling times, and free-energy estimator sophistication (plain MM-GBSA versus entropy-corrected or QM-refined variants) vary appreciably across the literature. This heterogeneity complicates direct cross-study comparison of predicted binding affinities and argues for standardized benchmarking when evaluating candidate PCSK9 modulators computationally.

#### 2.4.4. Clinical Informatics and Systems Biology

On the clinical-informatics, modern machine-learning models trained on large-scale biobank datasets are increasingly incorporating genetic information to enhance detection and risk stratification. For example, the FIND FH machine-learning model mined electronic health-encounter data from over 270 million individuals to flag probable familial hypercholesterolemia cases [108]. This underscores the potential value of incorporating genetic information regarding PCSK9 variant carriers to enhance detection and risk stratification for familial hypercholesterolemia and related dyslipidemias [109].

Finally, integrative genomic–proteomic methodologies, such as proteomics-informed Mendelian randomization with colocalization, provide context for situating PCSK9 within causal lipid networks and alongside other protein targets. This unified analytical approach establishes a comprehensive, systems-level framework, enabling seamless integration with transcriptomic data, proteomic data, and druggability evaluations to effectively prioritize genetic variants and modulators [110].

#### 2.4.5. Structural Gaps and the Pdb

While the methods discussed above highlight PCSK9’s overall role in disease pathways, structural biology asks a complementary question: how do specific substitutions physically alter the protein? A structure-first perspective on this begins by surveying the Protein Data Bank (PDB) itself. A recurring pattern across deposited PCSK9 structures is that the distal C-terminal CHRD (approximately residues 680–692) is frequently unresolved or deliberately truncated. PDB entry 4OV6 omits the CHRD entirely, apo structures such as 2P4E (1.98 Å) and 2PMW (2.30 Å) model only 581–586 of the 692 residues, and only Fab- or nanobody-stabilized structures (6U36, 6U38, 7ANQ) extend coverage to residues ~683. This recurring gap is itself informative, indicating that the CHRD C-terminal tail is intrinsically flexible rather than simply under-sampled. The AlphaFold model of Q8NBP7 [98] offers a complementary check, assigning low per-residue confidence (pLDDT < 70) to this same segment, alongside elevated Predicted Aligned Error relative to the structured core (69). This is consistent with the crystallographic evidence, though this is not fully independent corroboration, since AlphaFold is trained on Protein Data Bank depositions, its reduced confidence likely reflects the underlying scarcity of experimental structural coverage for this segment. For comprehensive variant interpretation, an integrative strategy that combines the highest-resolution rigid-core structure with AlphaFold-seeded molecular dynamics of the flexible tail is therefore preferable to relying on either source alone.

The AlphaFold-based analyses discussed above are confined to the PCSK9 monomer. The PCSK9–LDLR complexes examined computationally in this review (Section 2.1, Table 1) are still built from experimentally determined assemblies (PDB 3BPS, 2P4E, 3P5C) rather than AI-predicted ones. AlphaFold-Multimer extended the original network to co-fold interacting chains [90], and AlphaFold3 further generalized this to a diffusion-based architecture that jointly models proteins with ligands, ions, and covalently modified residues in a single prediction [96]. This latter capability is particularly relevant to the Fam20C-phosphorylated CHRD loop discussed in Section 3.3. To our knowledge, no published study has used AlphaFold3 or an equivalent joint co-folding architecture to predict this three-way assembly with the phosphorylated Ser666/Ser668 state modeled explicitly. As a preliminary step toward this goal, described in Section Authors’ Preliminary Interaction Analyses: The Pcsk9-Fam20c-Cap1 Ternary Complex, we assembled a representative PCSK9-FAM20C-CAP1 structural composite by docking independently AlphaFold-predicted components; this composite approximates, rather than resolves, the phosphorylation-dependent geometry of the interaction. Evaluating this interface requires modeling not only the phosphorylated serines but also the surrounding microenvironment, particularly the cluster of arginines critical for mediating these transient electrostatic interactions. Independent benchmarking indicates that AlphaFold3 improves on AlphaFold-Multimer for well-ordered interfaces, but that both remain comparatively less reliable for flexible or disordered regions [97]. This reinforces this section’s earlier point that intrinsically disordered segments, such as the CHRD M3 loop, are better modeled by combining a high-resolution experimental core with AI-seeded molecular dynamics than by static AI structure prediction alone.

This same AI-seeded logic extends naturally to pocket discovery. Several PCSK9 regulatory surfaces, including the CHRD, lack a classical orthosteric catalytic pocket. To overcome this, researchers can employ graph neural network methods trained on molecular-dynamics-derived pocket-opening events, such as PocketMiner [111], as well deep equivariant structure-prediction models capable of sampling induced-fit conformations. These approaches offer a route to flagging transient, druggable cavities that are invisible in any single static structure. If applied to PCSK9, cryptic-pocket detection of this kind could complement the pathogenicity- and MD-based workflows described above, potentially extending variant and therapeutic-target assessment to regulatory surfaces beyond the canonical LDLR-binding interface. Together, these structural and computational tools set the stage for the clinical and genetic discussion that follow. Accordingly, Section 3 introduces the naturally occurring genetic variants that map onto, and are mechanistically explained by, the domain architecture and dynamics described herein.

## 3. Genetic Variations in Pcsk9 and Hypercholesterolemia in Monogenic Disorders

### 3.1. Gain-of-Function (Gof) Variants

Mapped onto the domain architecture introduced in Section 2.1, GOF and LOF substitutions cluster non-randomly. GOF variants concentrate at the catalytic-domain/EGF-A interface and along the prodomain–catalytic-domain contact, where they can strengthen LDLR binding or slow the intracellular processing that would otherwise limit PCSK9 secretion. Conversely, LOF variants disproportionately affect the prodomain, the catalytic triad, or CHRD residues required for proper folding and trafficking, destabilizing the protein or preventing its exit from the secretory pathway altogether. This structural logic, position relative to the LDLR interface versus position relative to the folding/trafficking machinery, offers a working hypothesis for why a given substitution behaves as GOF or LOF, though it remains a general framework rather than a variant-by-variant rule.

GOF variants in the PCSK9 gene are a well-recognized cause of autosomal-dominant hypercholesterolemia, a monogenic disorder characterized by markedly elevated LDL-C and premature cardiovascular disease. The discovery of these variants established PCSK9 as a critical regulator of lipid metabolism and a central therapeutic target. Common GOF variants include D374Y, S127R, F216L, and R496W, which have been identified in families with severe hypercholesterolemia in the absence of variants in LDLR or APOB genes [32,39]. These variants increase the activity or stability of the PCSK9 protein, resulting in enhanced degradation of the LDLR and consequently higher circulating LDL-C levels [40].

As established earlier, the canonical role of secreted PCSK9 is to bind hepatocyte LDLR and route it to the lysosome, preventing its recycling to the cell surface and thereby limiting LDL-C uptake. Consistent with the structural framework above, GOF variants pathologically hyperactivate this baseline process by increasing PCSK9–LDLR affinity or favoring intracellular trafficking routes that accelerate receptor clearance [40]. For example, the D374Y variant significantly increases the binding affinity between PCSK9 and LDLR, producing a stronger and more persistent interaction that prevents receptor recycling. Structural analyses indicate that this variant alters the conformation of the catalytic domain, enhancing receptor binding and stabilizing the PCSK9–LDLR complex [38].

Clinically, individuals carrying GOF variants present with severe hypercholesterolemia from early life. Plasma LDL-C levels can exceed 300–400 mg/dL in some cases, and affected individuals often exhibit premature atherosclerotic cardiovascular disease, including myocardial infarction at a young age. Family histories frequently reveal multiple affected relatives, consistent with autosomal-dominant inheritance patterns. Studies demonstrate that the combination of GOF variants such as S127R and D374Y can have additive effects on PCSK9 activity, resulting in dramatically increased receptor degradation and reduced LDL uptake [37]. Overall, these variants exemplify how structural alterations in PCSK9 translate into significant functional and clinical consequences.

### 3.2. Loss-of-Function (Lof) Variants

As introduced above, LOF variants tend to follow the complementary structural logic. Rather than clustering at the LDLR-binding interface, they more often disrupt folding, catalytic maturation, or trafficking signals elsewhere in the protein.

In contrast to GOF variants, LOF variants in PCSK9 are associated with reduced LDL-C levels and a markedly decreased risk of cardiovascular disease. Common LOF variants include R46L, Y142X, and C679X, which impair PCSK9 synthesis, secretion, or function. These variants reduce the capacity of PCSK9 to bind and route LDLR to the lysosome, thereby preserving receptor recycling and resulting in increased receptor density on hepatocytes and enhanced clearance of circulating LDL-C [42].

The physiological relevance of LOF variants became evident through population studies showing that individuals carrying such variants exhibit significantly lower LDL-C concentrations and reduced incidence of coronary heart disease. Genetic evidence demonstrates a direct correlation between LOF variants and reduced plasma cholesterol levels, highlighting the causal role of PCSK9 in lipid regulation [40,41]. Importantly, these individuals generally do not display adverse clinical effects, indicating that partial or complete inhibition of PCSK9 is well tolerated.

From a structural perspective, LOF variants often destabilize the PCSK9 protein or disrupt its interaction with LDLR. For example, nonsense variants such as Y142X produce truncated proteins that are rapidly degraded, while missense variants like R46L reduce the affinity of PCSK9 for LDLR. These alterations diminish receptor degradation and favor increased LDL clearance. The protective cardiovascular phenotype associated with LOF variants has provided strong genetic validation for the development of PCSK9-targeted therapies, making this pathway one of the most successful examples of translational genomics in cardiovascular medicine [5,43]. Table 1 summarizes these major PCSK9 gain- and loss-of-function variants, detailing their domain locations, mechanistic categories, and associated clinical phenotypes.

Beyond these rare monogenic LOF variants, numerous common single nucleotide polymorphisms (SNPs) in PCSK9 have also been identified in the general population. Population-based sequencing studies have demonstrated a spectrum of PCSK9 alleles, ranging from rare high-impact variants to common polymorphisms that exert relatively small effects on plasma LDL-C levels [58]. These common variants typically produce modest reductions or increase in LDL-C and are associated with moderate changes in cardiovascular risk rather than the severe phenotypes observed in monogenic gain- or loss-of-function variants [112]. Large genome-wide association studies have further confirmed that PCSK9 polymorphisms contribute to lipid variability as part of a broader polygenic architecture, with each locus generally exerting only a small effect on cholesterol concentrations [113,114].

Because these individual polygenic effects are modest, their ultimate clinical impact is highly sensitive to gene–environment interactions. Gene–environment interactions play an important role in determining the clinical impact of these variants. Environmental factors such as diet, physical activity, and metabolic comorbidities can modulate the phenotypic expression of PCSK9 polymorphisms, influencing plasma lipid levels and cardiovascular risk [115]. For example, individuals carrying certain PCSK9 variants may exhibit different lipid responses depending on dietary fat intake or overall metabolic status, highlighting the interaction between genetic background and lifestyle factors. While these variants rarely cause monogenic hypercholesterolemia, they contribute to cumulative polygenic risk scores and may influence individual responses to lipid-lowering therapies.

### 3.3. E670g: A Case Study in CHRD Module M3 Regulation

Beyond the canonical GOF/LOF dichotomy described above, the common polymorphism E670G (p.Glu670Gly, rs505151) illustrates how structural and regulatory context, rather than a simple change in LDLR-binding affinity, can determine a variant’s clinical phenotype. E670 lies within CHRD module M3 (residues 604–692), one of three pseudo-symmetric, six-stranded β-sandwich modules (M1: 453–529; M2: 530–603; M3: 604–692) that adopt a fold structurally related to the adipokine resistin. While the rigid, disulfide-stabilized core of M3 is well resolved crystallographically, a short stretch centered on Ser666 is disordered in the deposited structures. In PDB 2P4E (chain A), residues 660–667 lack coordinates, while Ser668 and E670 itself are resolved. In PDB 2PMW, the missing stretch extends further, spanning residues 660–669 and thus also encompassing Ser668, while the flanking Arg657/Arg659 and residue 670 onward remain resolved. This localized, variable disorder around Ser666, rather than a broad unresolved region encompassing E670, is nonetheless consistent with a solvent-exposed, dynamic regulatory element immediately flanking two experimentally anchored, functionally annotated arginines. This loop is a substrate for the Golgi kinase Fam20C, which phosphorylates PCSK9 at Ser666, Ser668, and Ser688 via the S-X-E/pS recognition motif. Phosphorylation, particularly at Ser668 (motif Ser668-Ile-Glu670), enhances PCSK9 secretion and its capacity to route LDLR for lysosomal clearance [44]. The E670G substitution removes the glutamate required at the +2 position of this motif, abolishing efficient Fam20C phosphorylation at Ser668 and shifting residual phosphorylation toward Ser666. Importantly, the primary sequence flanking this motif is highly arginine-rich: Arg657 and Arg659 precede the Ser666/Ser668 phosphosites, while Arg680 and Arg682 follow position 670 (UniProt Q8NBP7). These basic residues likely contribute electrostatically to co-factor engagement or dictate the local conformational preferences of the M3 loop. Consistent with this, alanine substitution of Arg657 and Arg659 together with M1 Arg491 (triple mutant PCSK9-3RA) abolishes CHRD binding to CAP1 by bio-layer interferometry and sharply reduces PCSK9 activity on the LDLR, confirming these M3 arginines as direct CAP1-binding determinants rather than purely conformational contributors [61]. Phosphorylated CHRD exhibits enhanced binding to CAP1, a proposed “Protein X” co-factor that engages modules M1/M3 to help route the PCSK9–LDLR complex to the lysosome [61]. This sequential FAM20C-CAP1 engagement, and the CHRD conformational gating that governs it, is illustrated structurally in Figure 3. Consequently, the E670G variant paradoxically reduces CAP1 recruitment and phosphorylation-dependent activity in vitro [44,61]. Despite this loss of interaction, E670G is clinically classified as a mild gain-of-function variant associated with elevated LDL-C and increased coronary artery disease risk, particularly in East Asian cohorts [45].

Reconciling this apparent paradox (reduced phosphorylation and CAP1 binding in vitro versus a net gain-of-function phenotype in vivo) remains an active area of mechanistic hypothesis generation. Because the exact mechanism remains unestablished by direct experiment, we propose the following non-mutually exclusive hypotheses. Crucially, each of these is rooted in the atomic reality of the E670 microenvironment, providing a starting framework for future testing:

(i) Evasion of clearance: Because the M3 loop is highly solvent-exposed, it likely acts as a prominent recognition motif. The E670G substitution prevents the addition of a bulky, highly charged phosphate group at Ser668, potentially removing a critical turnover signal. Without this visible flag, the protein may evade clearance receptors, extending the circulating half-life of PCSK9. (ii) Creation of an electrostatic clamp: E670 is embedded in a dense microenvironment of basic residues. Replacing the negatively charged glutamate with a neutral glycine, while simultaneously preventing adjacent phosphorylation, transforms this flexible loop into a concentrated patch of positive charge. At acidic endosomal pH, this dynamic, basic loop could act as a tentacle, clamping onto negatively charged LDLR surfaces or endosomal membranes and preventing receptor recycling independently of CAP1. (iii) Allosteric disruption of regulatory partners: Although E670 is located in module M3, the CHRD is an allosterically coupled unit. The increased backbone flexibility introduced by the glycine substitution could alter the conformational ensemble of the entire CHRD. This structural shift, or the resulting steric hindrance, could disrupt the adjacent M1/M2 interfaces where endogenous inhibitors, such as Annexin A2, or alternative co-factors, such as the M2-binding HLA-C, normally engage. Losing this native inhibition or altering these secondary partnerships would shift the balance toward LDLR clearance despite the reduced recruitment of CAP1.

Because these hypotheses depend fundamentally on the conformational dynamics and electrostatic properties of the disordered M3 loop, molecular dynamics (MD) of the phosphorylated versus E670G M3 loop offers a highly targeted, comparatively low-cost first step. Ideally performed in complex with a CAP1 structural model, this MD-Based approach builds directly on the computational approaches noted in Section 2.3 and can directly evaluate the shifting electrostatic surface of the arginine-rich microenvironment. Suitable homology templates for such a complex already exist. The human CAP1 C-terminal CARP/dimerization domain (PDB 1K8F, residues 319–475), the mouse CAP1 helical-fold and CARP domains (PDB 6RSW/6FM2, ~90% sequence identity to human), and an additional C-terminal domain conformer from the yeast Cap ortholog (PDB 1KQ5, bearing an engineered P505S substitution) together span the SH3-binding-domain region implicated in PCSK9 binding. Together with the mammalian structures, this yeast conformer offers a useful alternative fold state for modeling rather than a direct human/mouse template. Indeed, the preliminary PCSK9-FAM20C-CAP1 composite reported in Section Authors’ Preliminary Interaction Analyses: The Pcsk9-Fam20c-Cap1 Ternary Complex and Figure 3 already provides a first-pass HADDOCK/AlphaFold2-Multimer-guided PCSK9-CHRD–CAP1 docking model built along these same lines. The outstanding gap is therefore not the docking step itself, but subjecting that static composite, with the phosphorylated Ser666/Ser668 state modeled explicitly rather than approximated, to the MD refinement proposed above. Accordingly, we included E670G in Table 1 as a variant whose gain-of-function phenotype operates through disrupted post-translational regulation and cofactor recruitment rather than direct alteration of catalytic-domain/LDLR affinity, mechanistically distinct from D374Y, S127R, F216L, and R496W.

## 4. Clinical Implications and Therapeutic Strategies

### 4.1. Genetic Screening and Diagnosis

Genetic testing for PCSK9 variants is formally recommended in patients with suspected familial hypercholesterolemia, particularly when LDL-C levels are markedly elevated or when there is a strong family history of premature cardiovascular disease. Identification of a pathogenic variant can confirm the diagnosis, guide treatment decisions, and enable cascade screening among relatives [116].

Interpretation of genetic results requires careful consideration of both variant type and functional impact. While GOF variants generally predict severe phenotypes and may warrant early, aggressive lipid-lowering therapy, the detection of LOF variants may indicate a lower cardiovascular risk profile. Integrating this genetic information into clinical care represents a key aspect of personalized medicine, allowing for risk stratification and tailored therapeutic strategies [117]. Importantly, this clinical interpretation increasingly draws on the structural evidence discussed throughout this review. The American College of Medical Genetics and Genomics and the Association for Molecular Pathology (ACMG/AMP) variant-classification framework explicitly permits computational and structural evidence, such as predicted destabilization or proximity to a functional interface, to support a pathogenic or benign classification. Consequently, the domain-mapping, ddG-prediction, and MD approaches detailed in Section 2.3 are not purely mechanistic exercises and can directly supply the same evidence codes required for diagnostic genetic reporting.

### 4.2. Pcsk9 Inhibitors

The discovery of PCSK9’s role in cholesterol metabolism led to the development of monoclonal antibodies targeting this protein. Two major agents, evolocumab [118] and alirocumab [119], bind circulating PCSK9 and prevent its interaction with LDLR. This molecular blockade mechanism preserves receptor recycling and increases hepatic clearance of LDL-C (Figure 1D). The structural basis for this blockade was established by earlier crystallographic work on precursor antibody Fabs. For instance, the CDR-H2 and CDR-H3 loops of an early antibody were shown to structurally mimic the LDLR EGF(A) domain, binding an epitope on the PCSK9 catalytic domain that overlaps the entire EGF(A)-binding surface [120]. Concurrently, a separate neutralizing Fab was co-crystallized with PCSK9 bound to an adjacent, LDLR-competitive epitope [121]. Together these structures established the general principle exploited by evolocumab and alirocumab, steric occlusion of the EGF(A)-binding surface rather than allosteric inhibition.

Clinical trials have demonstrated substantial LDL-C reductions of approximately 50–60% with these therapies [122,123]. In the FOURIER trial, evolocumab reduced LDL-C by about 59% and lowered major cardiovascular events by 15–20% [118]. Similarly, the ODYSSEY Outcomes trial showed significant LDL-C reductions and improved clinical outcomes with alirocumab [119]. These agents are particularly effective in patients with familial hypercholesterolemia, including those with GOF variants, as they directly counteract the excessive PCSK9 activity. Although evolocumab and alirocumab predate the modern computational-design era, their structural precedent now directly informs it. The epitope-mapping principles established for these first-generation antibodies are precisely what the epitope-directed generative design frameworks described in Section 2.3, such as RFantibody and related tools, aim to encode computationally. This structural foundation shortening the path from target epitope to candidate biologic for the next generation of anti-PCSK9 agents.

### 4.3. Safety and Side Effects of Pcsk9 Inhibition

Long-term clinical trials of PCSK9 inhibitors have demonstrated favorable safety profiles, with minimal adverse effects. Studies consistently show effective LDL-C reduction without significant increases in serious adverse events [124,125]. The absence of major safety concerns in individuals with natural LOF variants (as discussed in Section 3.2) provides lifelong genetic validation for the tolerability of profound PCSK9 inhibition.

Potential side effects include mild injection-site reactions and rare hypersensitivity responses. While ongoing long-term studies continue to evaluate possible off-target effects, particularly in relation to neurocognitive outcomes and metabolic parameters [118,126,127], current evidence firmly suggests that PCSK9 inhibition is both safe and effective for long-term cardiovascular risk reduction. This monitoring also extends to PCSK9’s own biology beyond LDL-C, given evidence that the protein can independently promote vascular inflammation and cardiomyocyte apoptosis, pyroptosis, and ferroptosis, mechanisms implicated in heart failure progression [128].

### 4.4. Emerging Therapies Targeting Pcsk9

In addition to monoclonal antibodies, new therapeutic strategies are being developed to inhibit PCSK9. One of the most advanced approaches is the use of small interfering RNA (siRNA), such as inclisiran [129]. This therapy targets hepatic PCSK9 mRNA, reducing protein synthesis and lowering LDL-C levels by approximately 40–50% in clinical trials [42,125] (Figure 1D). A pooled patient-level analysis across the ORION trial program confirmed sustained LDL-C reductions in both familial hypercholesterolemia and atherosclerotic cardiovascular disease patients with a favorable long-term safety profile [130], and a separate patient-level analysis of the phase 3 program reported a 26% lower probability of major adverse cardiovascular events with inclisiran, although this cardiovascular benefit remains hypothesis-generating pending a dedicated, blinded outcomes trial [131]. Inclisiran treatment offers the advantage of infrequent dosing, typically twice per year after initial loading doses.

Gene-editing technologies, including CRISPR-based approaches, are also being explored to create permanent reductions in PCSK9 expression. These strategies aim to replicate the protective effects observed in individuals with natural LOF variants. Future directions in PCSK9-targeted therapy include oral inhibitors, vaccines, and next-generation biologics [132] (Figure 1D). Computer-aided design is central to advancing every one of these modalities. For instance, structure-based virtual screening drives the discovery of oral small-molecule candidates, just as algorithmic guide-RNA design ensures the on-target precision of CRISPR base-editing agents.

However, it is within the context of next-generation biologics that computational tools present the most transformative opportunities. These modalities transcend traditional, bulky monoclonal antibodies, encompassing single-domain nanobodies (VHHs), bispecific binders, and highly stable de novo miniproteins. These formats offer distinct pharmacological advantages, including enhanced tissue penetration, lower immunogenicity, and the ability to access sterically restricted epitopes that full-sized antibodies cannot reach, such as the cryptic pockets within the CHRD. Computer-aided design and atomistic simulations are central to advancing each of these directions. While generative protein-design pipelines (Section 2.3) can propose epitope-targeted nanobody or miniprotein scaffolds ahead of wet-lab screening, static predictions alone are insufficient for drug development. Simulation engines present massive opportunities to refine computationally generated leads. For instance, MD simulations are required to validate the conformational stability of a designed nanobody’s hypervariable loops in explicit solvent. Furthermore, state-of-the-art enhanced sampling techniques, such as machine learning-guided adaptive sampling and deep Markov state models, can rigorously predict the binding kinetics and residence times of these smaller biologics against highly dynamic PCSK9 targets like the CHRD M3 loop. By coupling generative sequence design with AI-accelerated simulation and absolute binding free energy workflows, researchers can computationally mature next-generation biologics for optimal affinity and stability long before experimental synthesis.

As of 2026, several of these directions have reached clinical or regulatory maturity. On 16 July 2026 the FDA approved enlicitide decanoate (Lipfendra), a once-daily oral macrocyclic peptide that binds circulating PCSK9 and blocks its interaction with LDLR through the same mechanism as the injectable antibodies. This landmark decision makes it the first oral PCSK9 inhibitor approved by the FDA [36,133]. In the pivotal CORALreef Lipids and CORALreef HeFH phase 3 trials, enlicitide produced placebo-adjusted LDL-C reductions of 56–59% at 24 weeks. This efficacy is comparable to evolocumab and alirocumab, accompanied by a safety profile similar to a placebo [134]. Furthermore, a cardiovascular outcomes trial (CORALreef Outcomes) is ongoing, with results expected around 2029. A second oral candidate, the small molecule AZD0780 (laroprovstat), produced dose-dependent, placebo-adjusted LDL-C reductions of 35–50% over 12 weeks in the phase IIb PURSUIT trial. This enabled 84% of participants to reach guideline LDL-C targets, compared to only 13% on background statin therapy alone [135]. In parallel, one-time gene-editing approaches have progressed beyond the CRISPR concept noted above. For instance, VERVE-102, an in vivo base-editing medicine that inactivates PCSK9 in hepatocytes, produced dose-dependent PCSK9 reductions of up to 88% and LDL-C reductions of up to 62% after a single infusion in the phase 1b Heart-2 trial. These effects sustained for up to 18 months and no treatment-related serious adverse events reported [26]. The therapeutic has since received FDA Fast Track designation, and Phase 2 enrollment is planned. The arrival of an approved oral peptide, a late-stage oral small molecule, and a one-time gene-editing agent indicate that macrocyclic peptides, small molecules, and permanent gene-editing strategies are now clinically validated complements to the monoclonal-antibody and siRNA approaches described above. This momentum is occurring alongside continued patent activity covering peptide- and small-molecule PCSK9 ligands [136,137]. Each of these clinical successes was preceded by, and continues to rely on, the simulation and structure-prediction methods detailed in Section 2.3.

Looking beyond these clinically validated agents, the regulatory biology of the CHRD described in Section 2.3 and Section 3.3 points to a still largely unexploited axis of intervention: the domain’s cryptic allosteric surface and its phosphorylation-dependent cofactor interactions. Diffusion-based de novo design platforms such as RFdiffusion [104] can generate peptide or mini-protein binders conditioned on a specified epitope. This step is typically followed by sequence design with inverse-folding networks such as ProteinMPNN [102]; applied to PCSK9, such tools could in principle design short peptides that occlude the Ser668 Fam20C recognition motif. This would directly block the phosphorylation-dependent gain-of-function pathways without needing to target the primary catalytic interface. In parallel, the cryptic-pocket detection methods noted in Section 2.3 allow systematic scanning of the CHRD for transient small-molecule-binding cavities absent from any single static structure. An early discovery-stage report has proposed one such cryptic pocket in the PCSK9 C-terminal domain as a binding site for allosteric small-molecule modulators [138]. While this specific mechanism has not yet been established in peer-reviewed clinical data and should be treated as a hypothesis-generating lead rather than a validated target, it highlights the potential of CHRD to serve as an allosteric anchor for small-molecule intervention. A more rigorously validated example of allosteric small-molecule inhibition comes from a molecular-dynamics and mutual-information analysis of a tetrahydroisoquinoline compound series bound to a distinct allosteric site. The highest-affinity analogues (cpd6, cpd9) simultaneously engage Arg357 (catalytic domain), Arg458, and Arg476 (C-terminal domain) in a stable electrostatic network anchored by Asp360. Mutual-information-based dynamic mapping demonstrate that potent inhibitors preserve long-range dynamical coupling between this allosteric site and the LDLR-binding segment (Asp374–Cys378). In contrast, weak analogues fail to maintain this communication pathway [139].

Beyond structure-conditioned design, protein language models (PLMs) offer a complementary, sequence-only route to peptide-binder generation that does not require a pre-specified epitope structure. To illustrate, PepMLM fine-tunes the ESM-2 protein language model to generate short linear peptide binders directly from a target protein’s sequence. It utilizes a span-masking strategy that reconstructs a plausible binder region conditioned on the target. In silico benchmarking against AlphaFold-Multimer showed performance matching or exceeding RFdiffusion on structured targets. Furthermore, candidate peptides have been experimentally validated by fusing them to an E3 ubiquitin ligase domain to drive targeted degradation of disease-relevant proteins [105]. Applying this same peptide-PROTAC (Proteolysis Targeting Chimera) logic to PCSK9 offers a profound pharmacological advantage over conventional inhibitors, a strategy realized in practice by the Cadd4 degrader discussed below. While monoclonal antibodies strictly neutralize circulating, extracellular PCSK9, cell-permeable peptide degraders can intercept the target intracellularly. By physically tethering PCSK9 to the cellular ubiquitination machinery, these chimeric molecules trigger its destruction by the proteasome before secretion. Crucially, because the degrader is not consumed in the ubiquitination process, it operates catalytically rather than stoichiometrically, allowing a low concentration of drug to iteratively clear cellular PCSK9 pools. Concretely, Fan et al. recently realized this exact mechanistic paradigm. They designed and experimentally validated a computer-aided 10-mer peptide (Cadd4, sequence TSWEEYLDWV) that binds the PCSK9 catalytic domain and, fused to a VHL-recruiting ALAPYIP motif, this construct degrades both intracellular and secreted PCSK9 via the ubiquitin–proteasome pathway. This chimeric construct reduced hepatic PCSK9 by ~38% and plasma LDL-C by ~29% in a diet-induced hypercholesterolemia mouse model. This represents the first PCSK9-targeted degrader peptide with in vivo efficacy data, though its binder sequence was obtained by classical docking (HPEPDOCK, MDock) rather than a protein language model [106]. To our knowledge, no PLM-generated peptide binder targeting PCSK9 has yet been reported in the literature. Applying a target-sequence-conditioned PLM, such as PepMLM, directly to the PCSK9 sequence represents a concrete, presently untested proposal for generating a first-pass binder library. This generation could be seeded on either the LDLR-binding catalytic-domain surface or the FAM20C/Ser668 motif discussed in Section 3.3. The resulting library could then be triaged using the same AlphaFold-Multimer-based in silico filtering already validated for PepMLM, before progressing to the CADD-refinement and PROTAC-fusion pipeline successfully demonstrated for Cadd4.

A third design paradigm, distinct from both peptide-PROTAC degraders and neutralizing antibodies, uses PCSK9 itself, or fragments of it, as a vaccine antigen to elicit a durable polyclonal antibody response, avoiding the need for repeated biologic dosing. Structure-guided design has proven useful here too: computational optimization of a food-derived lupin peptide, guided by molecular modeling of its PCSK9-binding pose, previously yielded an analogue (P5-Best) with improved dual PCSK9/HMG-CoA reductase inhibitory activity, establishing that natural-peptide PCSK9 ligands can be systematically improved through structure-based design rather than found empirically [140]. More recently, this structure-guided logic has been extended to vaccine antigen design itself. Sun et al. integrated Protein Data Bank structures of PCSK9-antibody complexes with AlphaFold3 predictions to identify conformational B-cell epitopes, fusing the selected epitope to a heterologous T-helper sequence; the resulting vaccine induced durable anti-PCSK9 antibody titers across mice, guinea pigs, and rhesus macaques [141]. Independently, Malenšek et al. used AlphaFold2 and AlphaFold3 modeling to confirm that a chimeric PCSK9 antigen, engineered by transplanting solvent-exposed residues from a distantly related fish ortholog onto the human sequence to break self-tolerance while preserving the native fold, retained a conformation comparable to wild-type PCSK9 [142]. Fang et al. took a complementary nanoparticle-display approach, conjugating the D374Y catalytic domain to self-assembling 24-mer ferritin nanoparticles to increase antigen valency and immunogenicity [143]. Together, these studies indicate that structure-based computational design, whether applied to epitope selection, self-tolerance-breaking mutagenesis, or antigen-display architecture, is becoming as central to PCSK9 vaccine development as it already is to antibody and peptide-degrader design.

## 5. Conclusions and Future Directions

### 5.1. Summary of Key Findings

The structural and computational characterization of PCSK9 stands as a premier model for precision medicine, bridging the gap between genetic discovery and the treatment of hypercholesterolemia, a major global contributor to atherosclerotic cardiovascular disease. While human genetic studies demonstrated that specific variants lead to severe autosomal dominant hypercholesterolemia or confer marked cardioprotection, translating these findings into clinical practice required a profound structural understanding of how PCSK9 regulates LDL receptor (LDLR) degradation. High-resolution atomistic mapping ultimately provided the blueprint for current monoclonal antibodies and siRNA-based therapies, which have produced substantial cardiovascular benefits by altering receptor recycling and plasma LDL-C levels. However, moving beyond these first-generation therapies has required integrating experimental validation with advanced computational biology to capture the full mechanistic complexity of the protein.

Determining the precise impact of rare genetic substitutions on protein folding, secretion, and LDLR engagement necessitated atomistic characterization of conformational fluctuations and long-range dynamic coupling. Explicit-solvent molecular dynamics (MD) simulations revealed that regulatory competence is driven by dynamic conformational ensembles, particularly within the flexible loops of the subtilisin-like domain and the allosteric networks of the CHRD. Furthermore, advanced simulation methodologies have transformed our ability to target previously intractable surfaces. Mixed-solvent molecular dynamics and topological data analysis successfully uncovered transient, druggable cryptic pockets within the C-terminal domain. In parallel, mutual-information mapping characterized long-range allosteric communication pathways linking distal binding sites to the primary LDLR interface.

Crucially, these biophysical insights have converged with recent breakthroughs in artificial intelligence to drive the next generation of therapeutics (Supplementary Figure S1). The transition from classical rigid docking to AI-driven generative frameworks (including RFdiffusion, ProteinMPNN, and target-sequence-conditioned protein language models) enables the de novo design of nanobodies and peptide-PROTAC binders. However, as demonstrated throughout this review, generative sequence design remains dependent on rigorous physics-based validation. Coupling AI scaffolds with state-of-the-art enhanced sampling techniques, machine learning-guided adaptive sampling, and absolute binding free energy workflows provides the necessary resolution to evaluate conformational stability, binding kinetics, and loop dynamics long before experimental synthesis.

### 5.2. Unanswered Questions

Several open questions span both clinical and structural domains, highlighting specific methodological gaps in the current computational toolkit. For many rare PCSK9 variants identified through broad genetic screening, the three-dimensional consequences of the substitution remain uncharacterized. It is often unknown whether a specific mutation destabilizes the interface between the prodomain and the subtilisin-like domain, alters the CHRD binding surface, or changes proteolytic autoprocessing, leaving both pathogenicity and druggability uncertain. A major unanswered question is how to overcome current limitations in molecular dynamics to answer these structural unknowns. Standard MD protocols often lack the sampling timescales required to fully map highly disordered regions, such as the M3 loop, or to capture the transient opening of the CHRD’s cryptic allosteric networks. Furthermore, accurately simulating how post-translational modifications dynamically alter these conformational ensembles remains a significant computational hurdle.

Beyond simulation timescales, it remains unclear how reliably current deep-learning variant-effect predictors and classical force fields generalize across the full spectrum of naturally occurring PCSK9 variants, rather than just the handful of well-studied alleles used to train them. This computational gap directly impacts clinical understanding, as the precise atomic basis for allele-specific therapeutic responses has not been systematically mapped. For example, it is not fully understood why some LDLR-binding-interface variants are neutralized effectively by a given monoclonal antibody or siRNA while others exhibit structural resistance. Finally, the long-term physiological consequences of near-complete PCSK9 suppression remain unresolved at a mechanistic level. Because PCSK9 interacts with alternative, non-LDLR binding partners in various tissues [13], the structural interfaces mediating these pleiotropic effects must be mapped. Understanding these secondary interactions will dictate how selectively future structure-based therapeutics need to be designed to inhibit one systemic pathway while sparing others.

### 5.3. Future Research

Priority areas for future research follow directly from these structural gaps. Systematic structural and biophysical characterization of rare and understudied PCSK9 variants, incorporating techniques such as cryo-EM, crystallography, and molecular dynamics, would clarify their mechanistic effects and critically improve variant-effect prediction.

For the PCSK9-FAM20C-CAP1 composite introduced in Section Authors’ Preliminary Interaction Analyses: The Pcsk9-Fam20c-Cap1 Ternary Complex, both atomistic and coarse-grained MD represent natural, complementary validation strategies. Explicit-solvent, all-atom refinement, following the same GROMACS/CHARMM36m or AMBER/ff14SB protocols already applied elsewhere in this review to the PCSK9-LDLR interface [64,82,94,95], could test whether the HADDOCK-predicted FAM20C and CAP1 contacts (Tables S1 and S2) remain stable once the rigid-body docking pose is allowed to relax and equilibrate, and would allow the phosphorylated Ser666/Ser668 state to be modeled explicitly rather than approximated by an unphosphorylated proxy residue. Because the hinge-mediated transition between the closed and FAM20C/CAP1-accessible open CHRD conformations (Figure 3) was only approximated here by manual rigid-body rotation, and because such large-scale domain motions typically unfold on timescales beyond the reach of routine atomistic sampling, coarse-grained MD using a general-purpose force field such as Martini 3 [144] offers a complementary route to letting this conformational transition emerge dynamically rather than being imposed a priori, at a fraction of the computational cost of all-atom simulation. Coupling coarse-grained sampling of the open-closed equilibrium with atomistic backmapping and refinement of the resulting interface would provide a considerably more rigorous test of the FAM20C- and CAP1-binding hypotheses illustrated in Figure 3 than the present static composite allows.

Furthermore, structure-guided and generative approaches to therapeutic design must continue to evolve. Moving beyond foundational docking and MM-GBSA frameworks, the field must increasingly integrate the advanced computational methodologies now available. Applying AI-driven rational design, deep-learning structure prediction, and advanced free-energy calculations could yield inhibitors or stabilizers tailored to specific variant profiles rather than to wild-type PCSK9 alone. These computational tools will also be critical in guiding the next generation of gene-editing and gene-silencing strategies, ensuring that durable reductions in PCSK9 activity are safely matched to patient-specific genomic profiles.

Ultimately, the future of PCSK9 research relies on integrating structural bioinformatics, physics-based simulations, and machine learning into a unified computational pipeline. By connecting dynamic variant classification directly through to in silico candidate molecule generation, computational biology will provide the definitive framework necessary to translate the growing genetic catalog of PCSK9 variants into new, precisely targeted therapeutics.

## Data Availability

No new data were created or analyzed in this study. Data sharing is not applicable to this article.

## Supplementary Materials

The following supporting information can be downloaded at: https://www.mdpi.com/article/10.3390/ijms27219676/s1. Reference [145] is cited in the Supplementary Materials.

## Abbreviations

ACMG/AMP	American College of Medical Genetics and Genomics/Association for Molecular Pathology
AI	artificial intelligence
ASCVD	atherosclerotic cardiovascular disease
AUC	area under the receiver operating characteristic curve
CAD	coronary artery disease
CADD	computer-aided drug design
CAP1	cyclase-associated protein 1
CDR	complementarity-determining region
CHD	coronary heart disease
CHRD	C-terminal cysteine- and histidine-rich domain
CRISPR	clustered regularly interspaced short palindromic repeats
cryo-EM	cryo-electron microscopy
CTD	C-terminal domain
EGF-A	epidermal growth factor-like repeat A
ER	endoplasmic reticulum
FAM20C	family with sequence similarity 20, member C
FDA	U.S. Food and Drug Administration
FH	familial hypercholesterolemia
GOF	gain of function
HDL-C	high-density lipoprotein cholesterol
HNF-1α	hepatocyte nuclear factor-1α
IC50	half-maximal inhibitory concentration
LDL	low-density lipoprotein
LDL-C	low-density lipoprotein cholesterol
LDLR	low-density lipoprotein receptor
LOF	loss of function
MD	molecular dynamics
MM-GBSA	molecular mechanics generalized Born surface area
MM-PBSA	molecular mechanics Poisson–Boltzmann surface area
MSMD	mixed-solvent molecular dynamics
PCSK9	proprotein convertase subtilisin/kexin type 9
PDB	Protein Data Bank
PLM	protein language model
PROTAC	proteolysis-targeting chimera
QSAR	quantitative structure–activity relationship
RMSD	root-mean-square deviation
RSA	relative solvent accessibility
siRNA	small interfering RNA
SNP	single nucleotide polymorphism
SREBP-2	sterol regulatory element-binding protein 2
ΔΔG	change in protein folding free energy upon substitution
