# Supplementary material for: Emerging Computational Advances in Simulating PCSK9 Variant Dynamics for Therapeutic Design Against Hypercholesterolemia
Source: Int J Mol Sci. 2026 Oct 29;27(21):9676. doi: 10.3390/ijms27219676 (PMC13763288; doi:10.3390/ijms27219676)
Supplement: Supplementary file 1 [file ijms-27-09676-s001.zip › ijms-4572096-supplementary.pdf]

Supplementary Materials

Note S1. Table 1 annotations. LDL-C Effect key: ↑↑/↓↓ denote large (monogenic-scale) increases/decreases in plasma LDL-C; ↑/↓ denote moderate effects. E670G is categorized as GOF (mild) rather than a plain GOF because its mechanism, impaired Fam20C phosphorylation and CAP1 recruitment in vitro with a net gain-of-function phenotype in vivo (Section 3.3), does not fit the direct affinity-change mechanism used to classify the canonical monogenic variants. Citation note: most rows in Table 1 are additionally traceable to a dedicated primary discovery or functional-characterization study [47-49,52,54,55,57-60], cited alongside the curated compilations of refs. [12] and [46]. R218S and R357H are additionally traceable to Benjannet et al. [50] and/or Allard et al. [51]; N425S is traceable to Pisciotta et al. [53]. R468W is likewise traceable to Allard et al. [51], reported there as p.(R469W); we retain the R468W designation used throughout this review and by refs. [12] and [46], but flag this one-residue discrepancy pending confirmation of the numbering convention used in the original report. N157K carries a separate caveat: its gain-of-function classification follows its original description (Leren, 2004; [145]), in which the variant was found in a single patient who also carried the established gain-of-function variant D374Y on the other allele, such that N157K's own independent effect on LDL-C was never isolated; a later screening study (Berge et al., 2006; [57]) subsequently identified the same variant in a cohort ascertained for hypocholesterolemia and favorable statin response, and secondary compilations have listed N157K under both categories without resolving the discrepancy. We retain the gain-of-function classification here, consistent with the majority of the literature and its original discovery report, but flag this as an unresolved ambiguity rather than a settled finding.

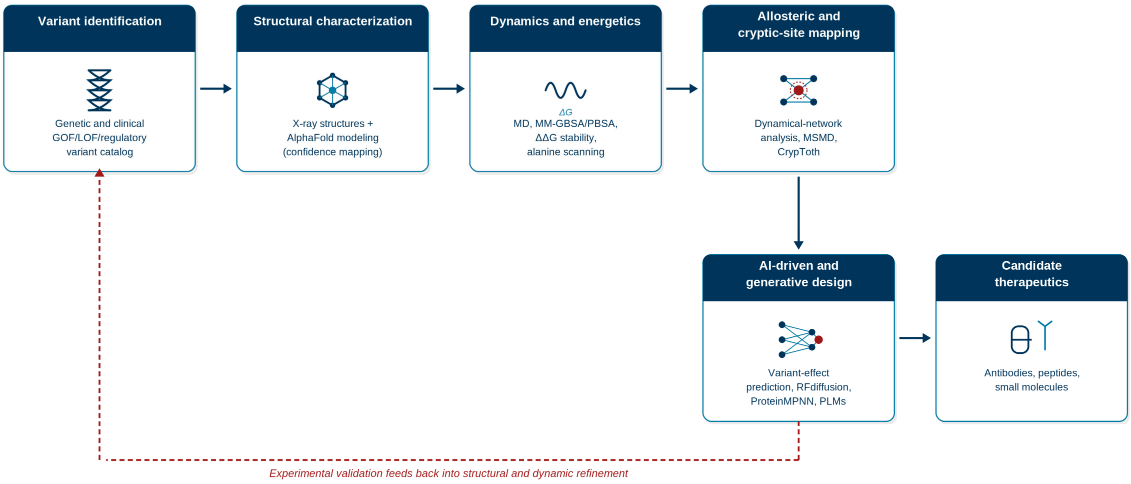

Figure S1. Computational workflow linking PCSK9 variant identification to therapeutic design.

Variant catalogs are structurally characterized by X-ray crystallography and AlphaFold modeling, then interrogated by molecular dynamics and free-energy methods and by dynamical-network and cryptic-pocket analysis, feeding into AI-driven and generative design of candidate antibody, peptide, and small-molecule therapeutics, whose experimental validation feeds back into structural and dynamic refinement (Table 2).

Table S1. PCSK9-FAM20C interface residue contacts (≤4.5 Å), from the best-scoring, most tightly converged HADDOCK2.4 cluster (HADDOCK score −115.97 ± 3.20; 25 members; intra-cluster RMSD 0.33 ± 0.20 Å). PCSK9 domain of origin given in parentheses (M2/M3 = CHRD subdomains). The Ser668 phosphoacceptor contacts FAM20C Asp458, Tyr411, His460, Thr268 and Arg408; additional CHRD M2 contacts (Asp570, Leu571, His574, Lys575, Pro576, Leu579, Arg580, Pro581) engage a separate FAM20C surface and likely reflect the permissive default passive-residue radius used in docking rather than a distinct second binding event.

PCSK9 residue	FAM20C residue	Dist. (Å)
Glu669 (M3)	Ser368	1.62
Ser666 (M3)	Arg408	1.68
Val661 (M3)	Tyr369	1.83
Leu579 (M2)	Ser106	1.91
Ser668 (M3)	Asp458	2.18
Asp367 (catalytic)	Lys2	2.25
Gln342 (catalytic)	Arg10	2.27
Thr667 (M3)	Arg408	2.45
His574 (M2)	Lys415	2.51
Gly665 (M3)	Arg408	2.64
Ser662 (M3)	Tyr369	2.66
Thr663 (M3)	Tyr369	2.72
Glu669 (M3)	Thr268	2.73
Arg580 (M2)	Glu108	2.86
Ser668 (M3)	Tyr411	3.08
Ser668 (M3)	His460	3.18
Arg580 (M2)	Ser106	3.20
Thr664 (M3)	Arg408	3.29
Pro616 (M3)	Ser368	3.35
Gly665 (M3)	Ser410	3.43
Pro581 (M2)	His105	3.48
Arg580 (M2)	His105	3.53
Pro618 (M3)	Ser368	3.57
Pro581 (M2)	Leu107	3.60
Ser668 (M3)	Thr268	3.62
Thr663 (M3)	Tyr411	3.78
Ser381 (catalytic)	Lys2	3.81
Ser668 (M3)	Arg408	3.92
Asp570 (M2)	Lys415	3.94
Asp660 (M3)	Lys413	4.07
Trp156 (catalytic)	Met3	4.25
Ser662 (M3)	Lys413	4.30
Pro576 (M2)	Arg408	4.32
Lys575 (M2)	Glu418	4.35
His574 (M2)	Ser410	4.40
Leu571 (M2)	Lys415	4.46

Table S2. PCSK9 CHRD-CAP1(SH3-binding domain) interface residue contacts (≤4.5 Å), from the top-ranked AlphaFold2-multimer/ColabFold model (rank 1 of 5; ipTM 0.181, pDockQ2 0.020) using the CAP1 SH3-binding linker (residues 214-337). CHRD domain of origin given in parentheses (M1/M3 = CHRD subdomains). Arg657 - one of three CHRD residues

(Arg491/Arg657/Arg659) whose combined mutation abolishes CAP1 binding by BLI (Fruchart-Gaillard et al. [61]) - makes

CHRD residue	CAP1 residue	Dist. (Å)
Arg458 (M1)	Glu323	1.67
Trp461 (M1)	Leu322	2.00
Thr459 (M1)	Leu324	2.03
Arg657 (M3)	Pro238	2.47
Val460 (M1)	Glu323	2.54
Arg458 (M1)	Glu325	2.61
Arg476 (M1)	Glu323	3.03
Trp461 (M1)	Pro234	3.18
Tyr648 (M3)	Pro237	3.23
Asp651 (M3)	Gly326	3.26
Gln621 (M3)	Pro238	3.28
Trp461 (M1)	Pro235	3.46
Arg458 (M1)	Leu324	3.58
Ser462 (M1)	Leu322	3.60
Glu481 (M1)	Glu325	3.85
Ala649 (M3)	Leu324	3.88
Val650 (M3)	Pro237	3.95
Val460 (M1)	Leu324	3.96
Ala478 (M1)	Glu323	4.05
Ala463 (M1)	Ala320	4.12
Cys477 (M1)	Glu323	4.21
Ala463 (M1)	Leu322	4.22
Tyr521 (M1)	Pro235	4.35

Supplementary Methods. Structural model construction for Figure 3 (main text) and Figures S1/Tables S1-S2 (below).

Model construction: the PCSK9 prodomain (residues 61-152) is from the crystal structure of the PCSK9 CHRD/P1.40 complex (PDB 3P5C); the catalytic domain, hinge, and CHRD (residues 153-682) are from a MODELLER v10.8 homology model (99.7% template identity; DOPE score −2964.4), renumbered to UniProt Q8NBP7 convention and cross-validated against an independent AlphaFold-predicted full-length PCSK9 chain (1.66-1.81 Å Cα RMSD across catalytic domain, hinge and CHRD; the homology model's own prodomain diverged from both the AlphaFold prediction and the 3P5C crystal structure, 7.66 Å, and was excluded in favor of the crystallographic coordinates). FAM20C (UniProt Q8IXL6) is from an AlphaFold-predicted PCSK9-FAM20C-CAP1 ternary complex, its isolated fold validated against the crystal structure of the *Caenorhabditis elegans* FAM20C ortholog (PDB 4KQA; 0.94 Å RMSD over a 264-residue conserved kinase-domain core), and docked onto PCSK9 with HADDOCK2.4's Easy Interface using PCSK9 Ser668 and FAM20C Asp478 as active residues (Asp478 identified via structural alignment to the catalytically essential Glu213 of the C. elegans ortholog; the deeply buried catalytic residue Glu306, RSA 5.8% by FreeSASA, was used as passive context). CAP1 (UniProt Q01518, SH3-binding linker residues 214-337) was predicted with AlphaFold2-multimer (ColabFold v1.6.2, default parameters) following the approach of Fruchart-Gaillard et al. [61] (Supplementary Figure 8), while the linker-restricted construct placed the literature-validated hotspot Arg657 in direct contact (2.47 Å). Because both partners require

the CHRD M1/M3 face solvent-exposed - occluded in the closed conformation adopted by PCSK9 in available crystal structures - an open conformation was approximated by rigidbody rotation of the CHRD (with bound partner) about the flexible hinge (residues 422- 452). All models are representative, illustrative composites; they have not been subjected to energy minimization or molecular dynamics refinement.
